# Supplementary material for: Depressive symptoms and workplace-violence-related risk factors among otorhinolaryngology nurses and physicians in Northern China: a cross-sectional study
Source: BMJ Open. 2018 Jan 27;8(1):e019514. doi: 10.1136/bmjopen-2017-019514 (PMC5829786; doi:10.1136/bmjopen-2017-019514)
Supplement: Author's manuscript [file bmjopen-2017-019514.draft_revisions.pdf]

BMJ Open

BMJ Open is committed to open peer review. As part of this commitment we make the peer review history of every article we publish publicly available.

When an article is published we post the peer reviewers' comments and the authors' responses online. We also post the versions of the paper that were used during peer review. These are the versions that the peer review comments apply to.

The versions of the paper that follow are the versions that were submitted during the peer review process. They are not the versions of record or the final published versions. They should not be cited or distributed as the published version of this manuscript.

BMJ Open is an open access journal and the full, final, typeset and author-corrected version of record of the manuscript is available on our site with no access controls, subscription charges or pay-per-view fees (http://bmjopen.bmj.com).

If you have any questions on BMJ Open's open peer review process please email editorial.bmjopen@bmj.com

BMJ Open

Depressive symptoms and workplace violence-related risk factors among otorhinolaryngology nurses and physicians in northern China: A cross-sectional study

Journal:	*BMJ Open*
Manuscript ID	bmjopen-2017-019514
Article Type:	Research
Date Submitted by the Author:	07-Sep-2017
Complete List of Authors:	Fang, Huiying; Harbin Medical University, Department of Health, Policy and Hospital Management, School of Public Health<br>Zhao, Xiao; Harbin Medical University School of Public Health, Department of Health Economy<br>Yang, Hai; the 2nd Affiliated Hospital of Harbin Medical University, Neurosurgery<br>Sun, Peihang; Harbin Medical University, Policy and Hospital Management<br>Li, Ying; Harbin Medical University, Policy and Hospital Management<br>Jiang, Ke; Harbin Medical University School of Public Health, Policy and Hospital Management<br>peng, li<br>Jiao, Mingli; Harbin Medical University, Health policy<br>Liu, Ming; the 2nd Affiliated Hospital of Harbin Medical University, Otorhinolaryngology<br>Qiao, Hong; the 2nd Affiliated Hospital of Harbin Medical University, Endocrine and Metabolic Diseases<br>Wu, Qunhong; Harbin Medical School, Social Medicine
**Primary Subject Heading**:	Public health
Secondary Subject Heading:	Ear, nose and throat/otolaryngology, Health policy
Keywords:	workplace violence, otorhinolaryngology, depressive symptoms, workplace violence-related risk factors.

SCHOLARONE™
Manuscripts

Depressive symptoms and workplace violence-related risk factors among otorhinolaryngology nurses and physicians in northern China: A cross-sectional study

Huiying Fang[1 †], Xiaowen Zhao[2 †], Haicheng Yang[3 †], Peihang Sun[4], Ying Li[5], Kexin Jiang[6], Peng Li[7], Mingli Jiao[8 *], Ming Liu[9 *], Hong Qiao[10 *], Qunhong Wu[11 *]

[1] Department of Health. Policy and Hospital Management, School of Public Health, Harbin Medical University, Harbin 150081, China; Email: 565453428@qq.com

[2] Department of Health Economy, School of Public Health, Harbin Medical University, Harbin 150081, China; Email: zhaoxiaowen12345@126.com

[3] Neurosurgery, the 2nd Affiliated Hospital of Harbin Medical University, Harbin 150081, China; Email: yanghaicheng123456@163.com

[4] Department of Health. Policy and Hospital Management, School of Public Health, Harbin Medical University, Harbin 150081, China; Email: 745450570@qq.com

[5] Department of Health. Policy and Hospital Management, School of Public Health, Harbin Medical University, Harbin 150081, China; Email: 1690342267@qq.com

[6] Department of Health. Policy and Hospital Management, School of Public Health, Harbin Medical University, Harbin 150081, China; Email: 1617358621@qq.com

[7] Department of Health. Policy and Hospital Management, School of Public Health, Harbin Medical University, Harbin 150081, China; Email: 656553133@qq.com

[8] Institute of Quantitative and Technical Economics, Chinese Academy of Social Science, Beijing 100000, China; Department of Health. Policy and Hospital

For peer review only - http://bmjopen.bmj.com/site/about/guidelines.xhtml

Management, School of Public Health, Harbin Medical University, Harbin 150081; Email: minglijiao@126.com

[9] Otorhinolaryngology, the 2nd Affiliated Hospital of Harbin Medical University, Harbin 150081, China; liuming6293@126.com

[10] Endocrine and Metabolic Diseases, the 2nd Affiliated Hospital of Harbin Medical University, Harbin 150081, China; qiaoh0823@sina.com

[11] Department of Social Medicine, School of Public Health, Harbin Medical University, Harbin 150081, China; qunhongwu@126.com

†These authors contributed equally to this work.

*Corresponding author: minglijiao@126.com (M.J.); liuming6293@126.com (M.L.); qiaoh0823@sina.com (H.Q.); qunhongwu@126.com (Q.W.); Tel.: +86-136-1367-5693 (M.J.); +86-131-0087-6901 (M.L.); +86-133-5986-4888 (H.Q.); +86-451-8750-2851 (Q.W.)

For peer review only - http://bmjopen.bmj.com/site/about/guidelines.xhtml

Abstract:

Objectives: Workplace physical violence among otorhinolaryngologists is relatively high among medical professions. This increased risk not only reduces the quality of provided medical services, but also increases the incidence of depression. Depressive symptoms among these physicians and nurses can seriously affect their job satisfaction, work efficiency, and overall happiness, resulting in a negative attitude toward providing treatment. We found few existing studies related to otorhinolaryngology physicians and nurses suffering from depression-related factors. Therefore, this study seeks to assess the level of depression in otorhinolaryngology physicians and nurses in northern China and to identify associations among depressive symptoms and workplace violence-related risk factors.

Methods: we conducted a cross-sectional study and make logistic regression analysis to investigate the relationship between workplace violence-related risk factors and depressive symptoms.

Results: Of the 652 participating otorhinolaryngologists and nurses in tertiary hospitals in Heilongjiang Province, China, 57.2% were found to have depressive symptoms, and of the respondents who had suffered from physical violence, 71.25% had depressive symptoms. Respondents who suffered from physical violence were more likely to suffer from depressive symptoms than those who had not.

Conclusions: This research indicates that the mental health of otorhinolaryngology physicians and nurses should be investigated to improve workplace relationships among physicians and nurses, as well as with their patients, and prevent the

For peer review only - http://bmjopen.bmj.com/site/about/guidelines.xhtml

occurrence of physical violence to reduce depressive symptoms.

Keywords: workplace violence; otorhinolaryngology; depressive symptoms; workplace violence-related risk factors.

Strengths and limitations of this study

1. The sample size of Grade A tertiary hospital in Heilongjiang Province may limit the study power.
2. The self-recall of the events of the past 12 months may underestimate the number of violent incidents.
3. Despite the limitations, this study can arouse managers' attention to the workplace violence that occur in the Otorhinolaryngology Department, reduce the occurrence of depressive symptoms of otorhinolaryngologists, and provide a guarantee for the improvement of medical service quality.

For peer review only

1. Introduction

In October 2013[1], a patient at the Zhejiang Provincial People's Hospital of Otorhinolaryngology stabbed to death the physician who was providing his diagnosis and treatment. Similarly, in February 2014, an otorhinolaryngologist at a hospital in Qiqihar City, Heilongjiang Province, China, was beaten to death during examination of a patient. In China, the frequency and severity of workplace violence among medical professionals has increased, as has its public attention [2]. Workplace violence (WPV) refers to the physical and psychological harm that professionals may face while performing their duties [3, 4]. The number of WPV incidents in health care facilities accounts for almost one-quarter of the total number of violent incidents in all workplace types [5], making healthcare workers the most vulnerable group to workplace violence [6]. The occurrence of hospital violence may affect the health of physicians and nurses and hinder their professional performance, resulting in a negative impact on patient services and overall health [7-9]. Differences in violence have been found across different departments in hospitals [10]; for example, many studies have focused on the high incidence of violence in emergency rooms [11, 12]; however, we found comparable rates of violent incidences in otorhinolaryngology departments. The China Hospital Association 2014 data show that from 2008 to 2012, the incidences of severe hospital violence increased from 47.7% to 63.7% [13]. Upon examining 30 cases of physicians and nurses who were victims of fatal WPV in China over the past 10 years, those working in otorhinolaryngology departments accounted for more than 10% of the total. The main causes of violence against

For peer review only

otorhinolaryngology physicians and nurses include (1) a lack of importance attached to otorhinolaryngology-related diseases by the patient; (2) physicians and nurses in this department have limited professional skills compared to those in other departments; and (3) treatment result of otorhinolaryngological diseases is not ideal [14]. Therefore, the working environment of otorhinolaryngology physicians and nurses should be a concern for hospital administrations, both from a mental health and WPV perspective.

Previous studies have shown that workplace violence is a contributing factor to employees suffering from mental illnesses, such as depression [15-17]. Further, the incidence of depression among adults in low- to middle-income countries was 11.1%, compared to 14.6% in high-income countries [18, 19]; specifically, the incidences of depression in the United States, Canada, and the UK was 12%, 19.3%, and 15.5%, respectively [20, 21]. In Canada, 1 in 10 nurses was found to have depressive symptoms [22], and in France the situation is even more serious, with one-third of nurse managers suffering from depressive symptoms [23]. Depressive symptoms can negatively affect job satisfaction, work efficiency, and overall happiness, resulting in a negative attitude toward providing medical treatment. Over the long term, this will not only affect the development of human resources in health fields, but also reduce the quality of medical care [8, 9, 24]. Related research shows that Chinese physicians and nurses experience different degrees of psychological issues, with the incidence of depression at 34.2% [25]. In southern China, Guangdong Province, depressive symptoms are associated with workplace violence, long working hours, shift

For peer review only - http://bmjopen.bmj.com/site/about/guidelines.xhtml

frequency, and departmental operations [7]. Previous studies reveal a difference in the proportion of depressive symptoms among nurses in different hospital departments; as such, managers should account for these differences among departments in their employee relations and processes [7]. Existing research primarily assesses the overall situation of a hospital, although few researches focus on specialized departments, primarily the emergency department. However, that the department of otolaryngology suffered a comparable amount of violent phenomena as emergency rooms and more than other departments; therefore, the occurrence of depressive symptoms among these staff should be taken seriously.

Otolaryngology is a key department in most hospitals, and has distinct differences from other departments. First, there are many types of otolaryngology-related diseases, each in unique anatomical locations, which affect patients' breathing, speech, smell, and hearing. Many such diseases have long cure times; therefore, providing patients with a fast and effective treatment is not always possible. Second, the patient population of an otolaryngology department can span a wide range of age groups, resulting in relatively large workloads for physicians and nurses. Third, otolaryngology involves a variety of specializations, requiring these physicians and nurses to engage in continual learning to improve their professional and technological skills [14, 26].

For these reasons, we hypothesized that the proportion of otolaryngology physicians and nurses suffering from depressive symptoms would be higher than that of other hospital departments. While some scholars have studied the influencing

For peer review only - http://bmjopen.bmj.com/site/about/guidelines.xhtml

factors of depressive symptoms among physicians and nurses in general, we found few previous studies on Chinese otorhinolaryngology physicians and nurses suffering from depression-related factors, including violence. Therefore, this study seeks to assess the level of depression in otorhinolaryngology physicians and nurses in northern China and to identify associations among depressive symptoms and workplace violence-related risk factors.

For peer review only

2. Methods

2.1. Study Population and Data Collection

A retrospective cross-sectional survey was conducted in Heilongjiang Province, which is located in northern China and has a population of 38.35 million. In the province, 63 Grade A tertiary hospitals are located in 13 cities. Due to the length of time allotted for this research and the limited time available to clinical staff., this study developed a web-based questionnaire approach to ensure the integrity and effectiveness of data collection using a convenience sampling method. First, with the help of the Heilongjiang Province physician's association otorhinolaryngology branch chairman, we obtained approval from the Heilongjiang Provincial Association of Otorhinolaryngology to conduct the study. The association then organized 9 experts to conduct a preliminary test of the questionnaire to ensure clarity, readability, and completeness of content. Second, the association provided a list of Grade A tertiary hospitals and their physicians' email contact information, from which we first contacted the otolaryngology director to seek help in getting the nurses' email addresses. We then sent an introductory email with a link to the informed consent form and questionnaire. After the initial e-mail was sent, a reminder e-mail was sent to those physicians and nurses who did not respond initially. Through this process, we obtained 652 valid questionnaires (total efficiency of 83.6%). The data were collected from January 10, 2017, to February 15, 2017.

2.2. Questionnaire

After obtaining written permission, we used a questionnaire developed by the

For peer review only - http://bmjopen.bmj.com/site/about/guidelines.xhtml

International Labor Office (ILO), International Council of Nurses (ICN), World Health Organization (WHO), and Public Service International (PSI) in 2003 to measure violence in the workplace [27]. We translated the questionnaire into Mandarin and then back into English to verify the accuracy of the Mandarin version. We then invited the aforementioned experts identified by the otorhinolaryngology association to modify the questions as needed, and then assessed the validity of the contents of the questionnaire, including the suitability for Chinese culture and appropriateness of translation. These experts included epidemiologists, health management specialists, public health specialists, health system specialists, otolaryngology experts, and administrative experts (15 persons). We conducted a two-week retest reliability check (Cronbach's α = 0.87) with a group of 42 otorhinolaryngology physicians and nurses from five Grade A hospitals and a second test after two weeks.

The final questionnaire included four parts: (1) demographic characteristics and workplace-related conditions; demographic characteristics included gender, age, years of experience, title, education level, marital status, and occupation, while workplace-related conditions included number of patients received per day, shift work, number of colleagues working together, and anxiety level from 1 (not at all) to 5 (extremely high); (2) situation of physical violence in the past 12 months (defined as intentional injury to the body); (3) situation of nonphysical violence in the past 12 months (including verbal abuse, threatening events, and sexual harassment); and (4) depressive symptoms, as measured by a Chinese version of the Zung Self-Rating

For peer review only - http://bmjopen.bmj.com/site/about/guidelines.xhtml

Depression Scale (SDS), which was able to reveal subjective feelings using 20 entries with a severity score ranging from 1 (very seldom presence of anxiety/depressive symptoms) to 4(anxiety/depressive symptoms are almost always present). The total score was recorded as the original score, then the scores of the 20 entries were added, and finally the resulting score was multiplied by 1.25 to get the standard score. Results with greater than or equal to 53 points were considered to have depressive symptoms [28, 29].

2.3. Data Analysis

Descriptive statistical calculations were conducted on demographic characteristics and frequency of depressive symptoms; in addition, the chi-test was used to analyse the correlation between symptoms of depression and the characteristics of the respondents as well as workplace violence-related factors. In addition, logistic regression analysis was used to determine which characteristics and factors (including years of experience, education level, title, number of patients received per day, how many colleagues work together, anxiety level, and physical violence) were likely to predict depression. SPSS v.19.0 was used for the analysis (statistical products and services solutions 19.0, 2010, IBM, Armonk, NY, USA). The value of $p < 0.05$ indicates statistical significance.

2.4. Ethics Statement

All respondents received an informed consent form, which included the goal of data collection and its use, the method of data collection, and consent for confidentiality. All respondents who agreed to the informed consent form then filled

For peer review only - http://bmjopen.bmj.com/site/about/guidelines.xhtml

out the questionnaire. The study protocol was reviewed and approved by the Research Ethics Committee of Harbin Medical University.

For peer review only

For peer review only - http://bmjopen.bmj.com/site/about/guidelines.xhtml

3.Results

Table 1 shows the demographic characteristics of the study participants. Of these otolaryngology staff, 63.7% were women, nearly half (41.7%) were between 30 and 39 years of age, and three-quarters (78.1%) were married. The majority of respondents (69.8%) had at least 10 years of experience in otolaryngology and 78.2% had a bachelor's degree or higher. Of the respondents, 19.5% held senior titles within the department.

Descriptive statistics associated with depressive symptoms of the participants are shown in Table 2, where 57.2% of the 652 otorhinolaryngology physicians and nurses had depressive symptoms. About half (49.54%) of the respondents worked with 1-2 colleagues more than half of their working hours. Further, of the respondents with depression symptoms, three quarters (76.41%) had experienced varying degrees of anxiety. Of the respondents who had suffered from physical violence, 71.25% had depressive symptoms. Associations between sample characteristics and depressive symptoms were assessed using Pearson chi-square tests and indicated that years of experience, title, number of colleagues more than half of their working hours, anxiety level, and physical violence were significantly associated with depressive symptoms.

Table 3 shows the adjusted odds ratio (OR) and 95% confidence interval (CI) of depressive symptoms, revealing that otorhinolaryngology physicians and nurses who suffered from physical violence (OR = 1.82, 95% CI = 1.06–3.12) were more likely to suffer from depressive symptoms than those who had not. Participants with less than 1 year of experience were more likely to suffer from depressive symptoms, while those

For peer review only - http://bmjopen.bmj.com/site/about/guidelines.xhtml

with more experience were progressively less likely to suffer from depressive symptoms. Otorhinolaryngology physicians and nurses who worked as the sole provider more than half of their working hours were more likely to suffer from depressive symptoms than those who worked frequently with other colleagues. Relative to the seniority of otorhinolaryngology physicians and nurses, formal staff and those with junior and intermediate titles showed depressive symptoms with protective factors.

For peer review only

4.Discussion

Our study examined the association of depressive symptoms and workplace violence-related risk factors for otorhinolaryngology physicians and nurses in northern China. Results confirm that depressive symptoms are common among professionals in this department, where the incidence of depressive symptoms was shown to be 57.2%. There is limited research on depression among otorhinolaryngology staff; hence, we could not directly compare our results with those of other studies. However, these results were significantly higher than the prevalence of depressive symptoms among general physicians in the United States (11.3%) [30] and the Netherlands (29%) [31]. The results in China were also higher than the prevalence of depressive symptoms among the US nurses (41%) [32] and Iranian physicians and nurses (28.76%) [33]. Moreover, this number is slightly higher than the incidence of depressive symptoms among emergency department nurses in Taiwan [1]. In addition, XF Yang and YJ Ji et al studies focused on China show that the incidence of depressive symptoms among healthcare workers across all hospital departments is 45.3% and 47.2%, respectively [34, 35]. The incidence of depression is also different among departments. Compared with other departments, otorhinolaryngology physicians and nurses have a higher incidence of depressive symptoms than most other departments outside of emergency and higher than normal levels [7, 36], though few other studies focus on the incidence of depressive symptoms among these professionals in China. In China, the otorhinolaryngology department is a significant and indispensable group within the hospital, as it is related

For peer review only - http://bmjopen.bmj.com/site/about/guidelines.xhtml

to patients' ear, nose, throat, and even the head and neck. Unfortunately, the frequency of violence within this department is relatively high due to various factors involving treatment options and professional skill levels [14]. Therefore, the research of association among depressive symptoms and workplace violence-related risk factors is essential for preserving not only the mental health of practitioners, but for ensuring quality of care.

This study found several factors associated with the occurrence of depressive symptoms. First, years of experience and title were correlated to the occurrence of depressive symptoms. The fewer the years of experience for physicians and nurses, the more likely they were to suffer from depressive symptoms. This has also been confirmed in previous studies on healthcare professionals in China [37]. Physicians and nurses with fewer years of experience often do not have enough social support and are more likely be affected by occupational stress [37, 38]. The mental state of physicians and nurses with few years of experience should be evaluated as early as possible to provide preventative and proactive psychological support as well as training opportunities [39].

Second, among WPV risk factors, the numbers of colleagues with whom a physician or nurse works the majority of the time was found to be a predictor of depression among these otorhinolaryngology professionals. Results of the survey show that compared with physicians and nurses who work with 2 colleagues most of the time, those who frequently work alone were more likely to suffer from depressive symptoms. Working alongside colleagues can help physicians and nurses deal with

For peer review only - http://bmjopen.bmj.com/site/about/guidelines.xhtml

pressure, dissatisfaction and other emotional fluctuations. Previous studies have also shown that social support can reduce the incidence of depressive symptoms, help individuals manage stress, and promote good mental health [40]. Peer support intervention, in particular, can effectively reduce depressive symptoms, as they not only can be done through daily interactions, but may also have the same effect as group cognitive behavioural therapy [41]. Therefore, the working status of medical staff (such as avoidance of working alone) should be considered to strengthen peer support and effectively prevent and improve depressive symptoms.

Third, this study revealed that physical violence is an important predictor of the incidence of depressive symptoms within the department of otolaryngology. Our findings suggest that the risk of depressive symptoms of physicians and nurses who have suffered physical violence is 1.82 times greater than those who have not. Previous surveys also indicate that workplace violence is a strong predictor of psychological problems such as depressive symptoms [15]. Depression is one of several negative yet likely consequences of physicians and nurses who suffer from workplace violence [42]. Among them, physical violence is more likely to cause physicians and nurses to experience depressive symptoms [39]. The reasons for the high rate of physical violence within otolaryngology departments are varied, as otorhinolaryngology-related diseases are often complex and involve longer courses of treatment, which can lead to patient insomnia, anxiety, and other psychological problems, leading to a loss of trust in their physicians and nurses. As such, these patients can be prone to adverse emotions [43]. Due to the nature of many

For peer review only

For peer review only - http://bmjopen.bmj.com/site/about/guidelines.xhtml

otorhinolaryngology diseases, which affect speech and hearing, patients are often unable to effectively express their dissatisfaction, making them more inclined to choose physical violence to express their discomfort or psychological distress [44]. Therefore, otorhinolaryngology physicians and nurses were found to suffer a higher frequency of severe physical violence (i.e., fatal or near-fatal attacks).

Physical violence can cause psychological damage to physicians and nurses as well. For example, it can increase the level of employee anxiety, resulting in poor productivity, lower quality of work, a decline in individual employee's sense of accomplishment, and poor relationships with patients, managers, and colleagues [11, 44, 45]. In addition, physical violence can also have a negative impact on physicians and nurses' social relationships both inside and outside the hospital [45]. Consequently, the relationship between physicians and nurses and their patients should be improved through different measures, including attaching importance to patients' psychological status during clinical diagnosis and treatment processes, enhancing the communication skills of otorhinolaryngology staff, and enhancing patients' awareness of the disease and potential treatment (including duration and adverse side-effects).

In previous studies of depressive symptoms in other hospital departments, the incidence of depressive symptoms was associated with physicians and nurses' workload and shift characteristics; however, this study did not find relevant results from these two factors among otorhinolaryngologists [46-49], though a study of resident physicians in Turkey is similar to the results of this study [50].

For peer review only - http://bmjopen.bmj.com/site/about/guidelines.xhtml

This study has the following limitations. First, we investigated only otorhinolaryngology physicians and nurses working in Grade A tertiary hospitals in Heilongjiang Province, so the results may not be fully representative of the entire situation in northern China and cannot be extended to other types of hospitals or departments. Future studies may also require in-depth exploration of the different types of otorhinolaryngological services at hospitals of different grades. Second, because the respondents were asked to review the events of the past 12 months, the recall process was unavoidably influenced by memory bias. Third, this study focuses on the more distinctive workplace violence-related factors for this analysis, though there may be additional factors related to the occurrence of depressive symptoms that could be further explored. Nevertheless, the current results suggest that otorhinolaryngology physicians and nurses are more susceptible to depressive symptoms than other healthcare professionals and that certain risk factors associated with workplace violence have a direct effect on this occurrence.

In summary, the results show that otorhinolaryngology physicians and nurses face a high risk of depressive symptoms in the Grade A tertiary hospitals in Heilongjiang Province, China. Of the 652 respondents, 57.2% had varying degrees of depressive symptoms. Our findings also show that workplace violence-related factors (years of experience, title, shifts worked with colleagues versus alone, and experiencing physical violence) were associated with symptoms of depression. Therefore, improving the relationships among physicians and nurses as well as their patients, focusing on the prevention or diffusion of physical violence, avoiding having the

For peer review only - http://bmjopen.bmj.com/site/about/guidelines.xhtml

physicians and nurses work alone, and bolstering support and training programs for staff with few years of experience are important to reduce depressive symptoms in otorhinolaryngology physicians and nurses. In view of our findings, the mental health of otorhinolaryngology physicians and nurses should be the focus for managers, to improve the service and morale within otolaryngology departments and seek additional root causes to reduce the occurrence of depressive symptoms.

For peer review only

References

1. HF Hsieh，YM Chen，HH Wang，SC Chang，SC Ma., Association among components of resilience and workplace violence - related depression among emergency department nurses in Taiwan: a cross - sectional study. Journal of Clinical Nursing, 2016. **25**(17-18): p. 2639-2647.
2. Qiang Ma, An Analysis of the Causes of Frequent Cases of Violent Injuries. National Medical Journal of China, 2014. 94(18): p. 1368-1370.
3. Healthcarefacilities, et al., Violence - Occupational hazards in hospitals. Hospitals, 2002.
4. Simpson, K.R., Incivility, Bullying, and Workplace Violence. Mcn the American Journal of Maternal/child Nursing, 2016. **41**(1): p. 68.
5. ISBN, Framework guidelines for addressing workplace violence in the health sector. Geneva Switzerland Ilo, 2002.
6. P Sun，X Zhang，Y Sun，H Ma,M Jiao, et al., Workplace Violence against Health Care Workers in North Chinese Hospitals: A Cross-Sectional Survey. International Journal of Environmental Research & Public Health, 2017. **14**(1).
7. Y Gong，T Han，X Yin，G Yang，R Zhuang, et al., Prevalence of depressive symptoms and work-related risk factors among nurses in public hospitals in southern China: A cross-sectional study. Scientific Reports, 2014. **4**: p. 7109.
8. CP Landrigan， JM Rothschild， JW Cronin， R Kaushal， E Burdick, et al., Effect of reducing interns' work hours on serious medical errors in intensive

For peer review only - http://bmjopen.bmj.com/site/about/guidelines.xhtml

care units. New England Journal of Medicine, 2004. **351**(18): p. 1838-1848.

9. SW Lockley，JW Cronin，EE Evans，BE Cade，CJ Lee, et al., Effect of reducing interns' weekly work hours on sleep and attentional failures. New England Journal of Medicine, 2004. **351**(18): p. 1829.

10. T Yan，S Li，G Pan，B Qu, et al., Perceived Risk of Violence in Various Hospital Levels and Departments in Urban and Rural China. Iranian Journal of Public Health, 2016. **45**(9): p. 1238-1240.

11. Eslamian, J., A.A. Akbarpoor, and S.A. Hoseini, Quality of work life and its association with workplace violence of the nurses in emergency departments. Iranian Journal of Nursing & Midwifery Research, 2015. **20**(1): p. 56-62.

12. Stene, J., Workplace violence in the emergency department: giving staff the tools and support to report. Permanente Journal, 2015. **19**(2): p. e113-7.

13. Lingling Wang, Chen Wang, Yanlin Cao, Yue Zhao, Yutao Cheng, Lili Zheng., Analysis and thoughts on trend and harm of hospital violence. Chinese Hospitals., 2014(3): p. 4-6.

14. Kai Zhang and Kang Xue, Difficulties and Countermeasures of Young Doctors in the Department of ENT in Primary Hospitals. Medicine and Law, 2016. 8(4): p. 45-47.

15. Aytaç, S. and S. Dursun, The effect on employees of violence climate in the workplace. Work, 2012. **41 Suppl 1**(6): p. 3026.

16. Merecz, D., M. Drabek, and A. Mościcka, Aggression at the workplace--psychological consequences of abusive encounter with coworkers

For peer review only - http://bmjopen.bmj.com/site/about/guidelines.xhtml

and clients. International Journal of Occupational Medicine & Environmental Health, 2009. **22**(3): p. 243.

17. ATCD Silva，MFT Peres，CDS Lopes，LB Schraiber，E Susser, et al., Violence at work and depressive symptoms in primary health care teams: a cross-sectional study in Brazil. Social Psychiatry and Psychiatric Epidemiology, 2015. **50**(9): p. 1-9.

18. Brundtland, G.H., Mental Health: New Understanding, New Hope. Journal of the American Medical Association, 2001. **286**(19): p. 2391-2391.

19. E Bromet，LH Andrade，I Hwang，NA Sampson，J Alonso, et al., Cross-national epidemiology of DSM-IV major depressive episode. BMC Medicine, 2011. **9**(1): p. 90-90.

20. S Coomber，C Todd，G Park，P Baxter，J Firthcozens, et al., Stress in UK intensive care unit doctors. Bja British Journal of Anaesthesia, 2002. **89**(6): p. 873.

21. Lloyd, S., D. Streiner, and S. Shannon, Burnout, depression, life and job satisfaction among Canadian emergency physicians. Journal of Emergency Medicine, 1994. **12**(4): p. 559-565.

22. Ohler, M.C., M.S. Kerr, and D.A. Forbes, Depression in nurses. The Canadian journal of nursing research = Revue canadienne de recherche en sciences infirmières, 2010. **42**(3): p. 66-82.

23. N Nourry，A Luc，F Lefebvre，H Sultantaïeb，S Béjean., Psychosocial and organizational work environment of nurse managers and self-reported

For peer review only - http://bmjopen.bmj.com/site/about/guidelines.xhtml

depressive symptoms: cross-sectional analysis from a cohort of nurse managers. International Journal of Occupational Medicine and Environmental Health, 2014. **27**(2): p. 252-69.

24. Xiaoying Zhang, Ji Gao, and Xuan Zhang, Correlation analysis about workplace violence and job burnout among the head nurses. Chinese Nursing Management, 2008. 8(3): p. 18-20.

25. Yanqin Yuan, Bo Wang, Xianni Wang, Kuihua Jin., Survey on Mental Health Status of the Medical Staff. Nursing Journal of Chinese People's Liberation Army, 2007. 24(12): p. 22-23.

26. Wei Li, Analysis of Psychological Characteristics of Patients with Otorhinolaryngology and Analysis of Psychological Nursing, Modern Diagnosis & Treatment, 2014(2): p. 1428-1429.

27. W Cai，L Deng，M Liu，M Yu., Antecedents of medical workplace violence in South China. Journal of Interpersonal Violence, 2010. **26**(2): p. 312-327.

28. Zhengyu Wang and Yufen Chi, Self-Rating Depression(SDS). Shanghai Archives of Psychiatry, 1984(2).

29. Zung, W.W.K., A Self-Rating Depression Scale. Archives of General Psychiatry, 1965. **12**(12): p. 63-70.

30. Schwenk, T.L., D.W. Gorenflo, and L.M. Leja, A survey on the impact of being depressed on the professional status and mental health care of physicians. Journal of Clinical Psychiatry, 2008. **69**(4): p. 617-20.

31. Ruitenburg, M.M., H.W. Frings-Dresen Monique, and J.K. Sluiter, The

For peer review only - http://bmjopen.bmj.com/site/about/guidelines.xhtml

prevalence of common mental disorders among hospital physicians and their association with self-reported work ability: a cross-sectional study. BMC Health Services Research, 2012. **12**(1): p. 1-7.

32. Ruggiero, J.S., Health, work variables, and job satisfaction among nurses. Journal of Nursing Administration, 2005. **35**(5): p. 254-263.

33. HR Roohafza，H Afshar，AH Keshteli，N Mohammadi，A Feizi, et al., What's the role of perceived social support and coping styles in depression and anxiety? Journal of Research in Medical Sciences the Official Journal of Isfahan University of Medical Sciences, 2014. **19**(10): p. 944-9.

34. Xiaofang Yang and Shaoyun Hu, Related Study between Social Support and Coping Style, and Anxiety and Depression of Nurse. China Journal of Health Psychology, 2011. 19(3): p. 300-301.

35. Yongjuan Ji, Lina He, Jing Hao, Guifang Kuang., Correlation study of occupational stress and mental health among medical staff, Occupation and Health, 2015. 31(24): p. 3393-3395.

36. Y Gong，T Han，W Chen，HH Dib，G Yang, et al., Prevalence of anxiety and depressive symptoms and related risk factors among physicians in China: a cross-sectional study. Plos One, 2014. **9**(7): p. e103242.

37. Lijia Tian, Occupational stress and its relationship to depressive symptoms and anxiety disotder among ophthalmologists in Shanghai.2013, Fudan University.

38. M Raffaelli，FCD Andrade，AR Wiley，O Sanchez-Armass，LL Edwards,

For peer review only - http://bmjopen.bmj.com/site/about/guidelines.xhtml

Stress, Social Support, and Depression: A Test of the Stress-Buffering Hypothesis in a Mexican Sample. Journal of Research on Adolescence, 2013. **23**(2): p. 283–289.

39. C Rayner., A postal survey of doctors' attitudes to becoming mentally ill [J]. Clinical Medicine, 2009, 9(4):327-332.

40. HF Hsieh，YM Chen，HH Wang，SC Chang，SC Ma., Association among components of resilience and workplace violence-related depression among emergency department nurses in Taiwan: a cross-sectional study. Journal of Clinical Nursing, 2016. **25**(17-18): p. 2639-2647.

41. Piette, J.D., Efficacy of peer support interventions for depression: a meta-analysis. General Hospital Psychiatry, 2011. **33**(1): p. 29.

42. Alexy, E.M. and J.A. Hutchins, Workplace violence: a primer for critical care nurses. Critical Care Nursing Clinics of North America, 2006. **18**(3): p. 305-12.

43. Yu Liu and Weiping He, Ethical Ｒeflections on Medical Dispute in the Department of Otorhinolaryngology. Chinese Medical Ethics, 2016. 29(5): p. 777-779.

44. GM Roldɑ́n，IC Salazar，L Garrido，JM Ramos., Violence at work and its relationship with burnout, depression and anxiety in healthcare professionals of the emergency services. Health, 2013. **05**(2): p. 193-199.

45. D Yildirim., Bullying among nurses and its effects. International Nursing Review, 2009. **56**(4): p. 504-511.

For peer review only - http://bmjopen.bmj.com/site/about/guidelines.xhtml

46. Lee, H.E., H.R. Kim, and J.S. Park, Work-related risk factors for workplace violence among Korean employees. Journal of Occupational Health, 2014. **56**(1): p. 12.

47. Virtanen, M. and M. Kivimäki, Saved by the bell: does working too much increase the likelihood of depression? Expert Review of Neurotherapeutics, 2012. **12**(5): p. 497-499.

48. Bannai, A. and A. Tamakoshi, The association between long working hours and health: a systematic review of epidemiological evidence. Scandinavian Journal of Work Environment & Health, 2014. **40**(1): p. 5-18.

49. Oliveira, F.P.D., et al., Symptoms of depression and intervening factors among nurses of emergency hospital services. Acta Paulista De Enfermagem, 2015. **28**(3): p. 209-215.

50. F Demir，P Ay，M Erbaş，M Ozdil，E Yaşar., The prevalence of depression and its associated factors among resident doctors working in a training hospital in Istanbul. Türk psikiyatri dergisi = Turkish journal of psychiatry, 2007. **18**(1): p. 31.

Acknowledgments:

This study was funded by the Natural Science Foundation of China (Grant No. 71273002, 71473064， 71673073); New Century Excellent Talents of University from the Ministry of Education, China (Grant No.1252-NCET02); the China Postdoctoral Science Foundation (2015M570211, 2016T90181); the Heilongjiang Provincial

For peer review only - http://bmjopen.bmj.com/site/about/guidelines.xhtml

Association of Social Sciences (15058), and the Collaborative Innovation Centre of Social Risks Governance in Health.

Author Contributions Statement:

H.F., X.Z., H.Y. drafted the manuscript; J.M. designed the study; P.S., Y.L., K.J., and P.L. collected the data; J.M., M.L., H.Q., and Q.W. analyzed the data; J.M. contributed to the manuscript's revision. All authors approved the final manuscript for publication.

Conflict of interest declaration: The authors declare no conflict of interest

Data sharing statement: Data are available from the corresponding author upon request.

For peer review only

Table 1. Participant demographic characteristics (N = 652).

Characteristic	n	%
**Gender**		
Male	237	36.3%
Female	415	63.7%
**Age**		
≤29	214	32.8%
30–39	272	41.7%
≥40	166	25.5%
**Marital status**		
Unmarried	130	19.9%
Married	509	78.1%
Divorced	9	1.4%
Widowed	1	0.2%
Other	3	0.5%
**Years of experience**		
<1	76	11.7%
1-10	379	58.1%
11-20	126	19.3%
21-30	61	9.4%
>30	10	1.5%
**Title**		

For peer review only

For peer review only - http://bmjopen.bmj.com/site/about/guidelines.xhtml

Senior	127	19.5%
Intermediate	237	36.3%
Primary	168	25.8%
No title (formal doctor)	65	10.0%
Interns (resident training in hospital or post-graduate)	55	8.4%
**Level of education**		
Graduate and above	130	19.9%
Undergraduate	380	58.3%
Community college	97	14.9%
Vocational school	40	6.1%
Other	5	0.8%
**Occupation**		
Physician	379	58.1%
Nurse	273	41.9%

For peer review only

For peer review only - http://bmjopen.bmj.com/site/about/guidelines.xhtml

Table 2. Descriptive statistics associated with depressive symptoms of the participants.

Variables	Non-depressive tendency N = 279(42.8%) n (%)	Depressive tendency N = 373 (57.2%) n (%)	x^2	P
**Gender**			0.01	0.92
Male	102 (36.56%)	135 (36.19%)		
Female	177 (63.44%)	238 (63.81%)		
**Age**				
≤29	94 (33.69%)	120 (32.17%)	0.20	0.91
30–39	114 (40.86%)	158 (42.36%)		
≥40	71 (25.45%)	95 (25.47%)		
**Marital status**			4.92	0.30
Unmarried	49 (17.56%)	81 (21.72%)		
Married	226 (81.00%)	283 (75.88%)		
Divorced	2 (0.72%)	7 (1.88%)		
Widowed	0 (0%)	1 (0.27%)		
Other	2 (0.72%)	1 (0.27%)		
**Years of experience**			10.26	0.04
<1	33 (11.83%)	43 (11.53%)		
1-10	163 (58.42%)	216 (57.91%)		
11-20	48 (17.20%)	78 (20.91%)		

For peer review only - http://bmjopen.bmj.com/site/about/guidelines.xhtml

21-30	26 (9.32%)	35 (9.38%)		
>30	9 (3.23%)	1 (0.27%)		
**Title**			14.00	0.01
Senior	52 (18.64%)	75 (20.11%)		
Intermediate	89 (31.90%)	148 (39.68%)		
Primary	69 (24.73%)	99 (26.54%)		
No title (formal doctor)	39 (13.98%)	26 (6.97%)		
Interns (resident training in hospital or post-graduate)	30 (10.75%)	25 (6.70%)		
**Level of education**			7.32	0.12
Graduate and above	52 (18.64%)	78 (20.91%)		
Undergraduate	174 (62.37%)	206 (55.23%)		
Community college	31 (11.11%)	66 (17.69%)		
Vocational school	20 (7.17%)	20 (5.36%)		
Others	2 (0.72%)	3 (0.80%)		
**Occupation**			0.45	0.50
Physicians	158(56.63%)	221(59.25%)		
Nurses	121(43.37%)	152(40.75%)		
**Number of patients received per day**			1.02	0.91
1-20	122 (43.73%)	154 (41.29%)		

For peer review only - http://bmjopen.bmj.com/site/about/guidelines.xhtml

21-30	83 (29.75%)	116 (31.10%)		
31-40	39 (13.98%)	55 (14.75%)		
41-50	13 (4.66%)	22 (5.90%)		
>51	22 (7.89%)	26 (6.97%)		
**Shift work**			0.01	0.92
Yes	222 (79.57%)	298 (79.89%)		
No	57 (20.43%)	57 (15.28%)		
**Number of colleagues during work for more than half of the working hours**			13.62	0.01
A person	17 (6.09%)	40 (10.72%)		
2	107 (38.35%)	159 (42.63%)		
3-5	97 (34.77%)	129 (34.58%)		
6-10	35 (12.54%)	32 (8.58%)		
>10	23 (8.24%)	13 (3.49%)		
**Anxiety level**			9.54	0.04
Zero	89 (31.90%)	88 (23.59%)		
Low	52 (18.64%)	61 (16.35%)		
Moderate	74 (26.52%)	105 (28.15%)		
High	23 (8.24%)	38 (10.19%)		
Extremely high	41 (14.70%)	81 (21.72%)		

For peer review only

For peer review only - http://bmjopen.bmj.com/site/about/guidelines.xhtml

**Physical Violence**			3.34	0.01
Yes	23 (8.24%)	57 (15.28%)		
No	256 (91.76%)	316 (84.72%)		
**Non-Physical Violence**			3.43	0.06
Yes	91 (32.62%)	148 (39.68%)		
No	188 (67.38%)	225 (60.32%)		

For peer review only

Table 3. Multiple logistic regression of depressive and non-depressive symptoms.

	OR	95% CI	P
**Years of experience**			
<1	1.00	Reference	
1-10	0.04	0.01–0.39	0.01
11-20	0.06	0.01–0.52	0.01
21-30	0.06	0.01–0.52	0.01
>30	0.07	0.01–0.66	0.02
**Level of education**			
Graduate and above	1.00	Reference	
Undergraduate	1.32	0.20–8.89	0.78
Community college	1.38	0.21–8.85	0.74
Vocational school	0.67	0.10–4.46	0.68
Others	1.45	0.21–10.13	0.71
**Title**			
Senior	1.00	Reference	
Intermediate	0.37	0.16–0.85	0.02
Primary	0.35	0.17–0.73	0.01
No title (formal doctor)	0.48	0.23–0.99	0.04
Interns (resident training in hospital or	0.92	0.40–2.15	0.85

For peer review only - http://bmjopen.bmj.com/site/about/guidelines.xhtml

post-graduate)			
**Number of patients received per day**			
1-20	1.00	Reference	
21-30	1.03	0.52–2.06	0.93
31-40	0.98	0.48–1.98	0.94
41-50	0.88	0.40–1.93	0.75
>51	0.71	0.27–1.87	0.49
**Number of colleagues during work for more than half of the working hours**			
A person	1.00	Reference	
2	0.27	0.11–0.70	0.01
3-5	0.41	0.20–0.89	0.02
6-10	0.41	0.19–0.91	0.03
>10	0.73	0.30–1.74	0.47
**Anxiety level**			
Zero	1.00	Reference	
Low	1.84	1.09–3.09	0.02
Moderate	1.61	0.91–2.83	0.10

For peer review only - http://bmjopen.bmj.com/site/about/guidelines.xhtml

High	1.30	0.78–2.15	0.31
Extremely high	1.13	0.58–2.21	0.72
**Physical Violence**			
Yes	1.82	1.06–3.12	0.03
No	1.00	Reference	

For peer review only

STROBE 2007 (v4) Statement—Checklist of items that should be included in reports of *cross-sectional studies*

Section/Topic	Item #	Recommendation	Reported on page #
**Title and abstract**	1	(*a*) Indicate the study's design with a commonly used term in the title or the abstract	1
		(*b*) Provide in the abstract an informative and balanced summary of what was done and what was found	3-4
**Introduction**			
Background/rationale	2	Explain the scientific background and rationale for the investigation being reported	5-7
Objectives	3	State specific objectives, including any prespecified hypotheses	7-8
**Methods**			
Study design	4	Present key elements of study design early in the paper	9
Setting	5	Describe the setting, locations, and relevant dates, including periods of recruitment, exposure, follow-up, and data collection	9-11
Participants	6	(*a*) Give the eligibility criteria, and the sources and methods of selection of participants	9
Variables	7	Clearly define all outcomes, exposures, predictors, potential confounders, and effect modifiers. Give diagnostic criteria, if applicable	11
Data sources/ measurement	8*	For each variable of interest, give sources of data and details of methods of assessment (measurement). Describe comparability of assessment methods if there is more than one group	9-11
Bias	9	Describe any efforts to address potential sources of bias	*9-10*
Study size	10	Explain how the study size was arrived at	9
Quantitative variables	11	Explain how quantitative variables were handled in the analyses. If applicable, describe which groupings were chosen and why	9-11
Statistical methods	12	(*a*) Describe all statistical methods, including those used to control for confounding	11
		(*b*) Describe any methods used to examine subgroups and interactions	11
		(*c*) Explain how missing data were addressed	9
		(*d*) If applicable, describe analytical methods taking account of sampling strategy	9
		(*e*) Describe any sensitivity analyses	
**Results**			

For peer review only

Participants	13*	(a) Report numbers of individuals at each stage of study—eg numbers potentially eligible, examined for eligibility, confirmed eligible, included in the study, completing follow-up, and analysed	13/29-30
		(b) Give reasons for non-participation at each stage	
		(c) Consider use of a flow diagram	
Descriptive data	14*	(a) Give characteristics of study participants (eg demographic, clinical, social) and information on exposures and potential confounders	13/31-34
		(b) Indicate number of participants with missing data for each variable of interest	13
Outcome data	15*	Report numbers of outcome events or summary measures	13
Main results	16	(*a*) Give unadjusted estimates and, if applicable, confounder-adjusted estimates and their precision (eg, 95% confidence interval). Make clear which confounders were adjusted for and why they were included	13-14/35-37
		(*b*) Report category boundaries when continuous variables were categorized	35-37
		(*c*) If relevant, consider translating estimates of relative risk into absolute risk for a meaningful time period	
Other analyses	17	Report other analyses done—eg analyses of subgroups and interactions, and sensitivity analyses	
**Discussion**			
Key results	18	Summarise key results with reference to study objectives	15-16
Limitations	19	Discuss limitations of the study, taking into account sources of potential bias or imprecision. Discuss both direction and magnitude of any potential bias	19
Interpretation	20	Give a cautious overall interpretation of results considering objectives, limitations, multiplicity of analyses, results from similar studies, and other relevant evidence	15-18
Generalisability	21	Discuss the generalisability (external validity) of the study results	19-20
**Other information**			
Funding	22	Give the source of funding and the role of the funders for the present study and, if applicable, for the original study on which the present article is based	27-28

*Give information separately for cases and controls in case-control studies and, if applicable, for exposed and unexposed groups in cohort and cross-sectional studies.

Note: An Explanation and Elaboration article discusses each checklist item and gives methodological background and published examples of transparent reporting. The STROBE checklist is best used in conjunction with this article (freely available on the Web sites of PLoS Medicine at http://www.plosmedicine.org/, Annals of Internal Medicine at http://www.annals.org/, and Epidemiology at http://www.epidem.com/). Information on the STROBE Initiative is available at www.strobe-statement.org.

For peer review only

BMJ Open

Depressive symptoms and workplace violence-related risk factors among otorhinolaryngology nurses and physicians in northern China: A cross-sectional study

Journal:	*BMJ Open*
Manuscript ID	bmjopen-2017-019514.R1
Article Type:	Research
Date Submitted by the Author:	06-Nov-2017

Complete List of Authors:	Fang, Huiying; Harbin Medical University, Department of Health, Policy and Hospital Management, School of Public Health<br>Zhao, Xiao; Harbin Medical University School of Public Health, Department of Health Economy<br>Yang, Hai; the 2nd Affiliated Hospital of Harbin Medical University, Neurosurgery<br>Sun, Peihang; Harbin Medical University, Policy and Hospital Management<br>Li, Ying; Harbin Medical University, Policy and Hospital Management<br>Jiang, Ke; Harbin Medical University School of Public Health, Policy and Hospital Management<br>peng, li<br>Jiao, Mingli; Harbin Medical University, Health policy<br>Liu, Ming; the 2nd Affiliated Hospital of Harbin Medical University, Otorhinolaryngology<br>Qiao, Hong; the 2nd Affiliated Hospital of Harbin Medical University, Endocrine and Metabolic Diseases<br>Wu, Qunhong; Harbin Medical School, Social Medicine

**Primary Subject Heading**:	Public health
Secondary Subject Heading:	Ear, nose and throat/otolaryngology, Health policy
Keywords:	workplace violence, otorhinolaryngology, depressive symptoms, workplace violence-related risk factors.

SCHOLARONE™ Manuscripts

Depressive symptoms and workplace violence-related risk factors among otorhinolaryngology nurses and physicians in northern China: A cross-sectional study

Huiying Fang[1 †], Xiaowen Zhao[2 †], Haicheng Yang[3 †], Peihang Sun[4], Ying Li[5], Kexin Jiang[6], Peng Li[7], Mingli Jiao [8 *], Ming Liu[9 *], Hong Qiao[10 *], Qunhong Wu[11 *]

[1] Department of Health. Policy and Hospital Management, School of Public Health, Harbin Medical University, Harbin 150081, China; Email: 565453428@qq.com

[2] Department of Health Economy, School of Public Health, Harbin Medical University, Harbin 150081, China; Email: zhaoxiaowen12345@126.com

[3] Neurosurgery, the 2nd Affiliated Hospital of Harbin Medical University, Harbin 150081, China; Email: yanghaicheng123456@163.com

[4] Department of Health. Policy and Hospital Management, School of Public Health, Harbin Medical University, Harbin 150081, China; Email: 745450570@qq.com

[5] Department of Health. Policy and Hospital Management, School of Public Health, Harbin Medical University, Harbin 150081, China; Email: 1690342267@qq.com

[6] Department of Health. Policy and Hospital Management, School of Public Health, Harbin Medical University, Harbin 150081, China; Email: 1617358621@qq.com

[7] Department of Health. Policy and Hospital Management, School of Public Health,

For peer review only - http://bmjopen.bmj.com/site/about/guidelines.xhtml

Harbin Medical University, Harbin 150081, China; Email: 656553133@qq.com

[8] Institute of Quantitative and Technical Economics, Chinese Academy of Social Science, Beijing 100000, China; Department of Health. Policy and Hospital Management, School of Public Health, Harbin Medical University, Harbin 150081; Email: minglijiao@126.com

[9] Otorhinolaryngology, the 2nd Affiliated Hospital of Harbin Medical University, Harbin 150081, China; liuming6293@126.com

[10] Endocrine and Metabolic Diseases, the 2nd Affiliated Hospital of Harbin Medical University, Harbin 150081, China; qiaoh0823@sina.com

[11] Department of Social Medicine, School of Public Health, Harbin Medical University, Harbin 150081, China; qunhongwu@126.com

†These authors contributed equally to this work.

*Corresponding author: minglijiao@126.com (M.J.); liuming6293@126.com (M.L.); qiaoh0823@sina.com (H.Q.); qunhongwu@126.com (Q.W.); Tel.: +86-136-1367-5693 (M.J.); +86-131-0087-6901 (M.L.); +86-133-5986-4888 (H.Q.); +86-451-8750-2851 (Q.W.)

Word count: 3,691

For peer review only

For peer review only - http://bmjopen.bmj.com/site/about/guidelines.xhtml

For peer review only

Abstract

Objectives: Workplace violence is relatively frequent among medical professionals who work in otorhinolaryngology units. This phenomenon not only reduces the quality of provided medical care, but also increases the incidence of depressive symptoms among physicians and nurses, seriously affecting their job satisfaction and work efficiency with a negative attitude toward providing treatment. Few existing studies have assessed workplace-violence-related factors associated with depressive symptoms among otorhinolaryngology physicians and nurses.

Methods: We conducted a cross-sectional study in Grade A tertiary hospitals of

For peer review only - http://bmjopen.bmj.com/site/about/guidelines.xhtml

Heilongjiang Province in northern China, to evaluate the occurrence and level of depressive symptoms among otorhinolaryngology physicians and nurses and to analyse the relationship between them and workplace-violence-related risk factors and demographic variables.

Results: Of all our participating professionals, (379 otorhinolaryngologists and 273 nurses), 57.2% were found to have depressive symptoms, whereas, of the respondents who had suffered from physical violence, 71.25% had depressive symptoms. Professionals with less than one year of experience, as well as professionals who more frequently worked alone, were more likely to suffer from depressive symptoms than their colleagues.

Conclusions: This research addresses an emerging issue of clinical practice, and its results differ from those of previous studies; specifically, it indicates that the frequency of depressive symptoms among otorhinolaryngology physicians and nurses may be influenced by physical violence, the number of co-workers they have for more than half of their working hours, and other workplace-violence-related factors. To reduce the depressive symptoms caused by workplace violence and improve the quality of medical services, medical institutions should implement effective measures to prevent the occurrence of physical violence, strengthen team cooperation ability, and increase peer support.

Keywords: Workplace violence; otorhinolaryngology; depressive symptoms;

For peer review only - http://bmjopen.bmj.com/site/about/guidelines.xhtml

workplace violence-related risk factors.

STRENGHTS AND LIMITATIONS OF THIS STUDY

1. The sample size, which is based on the number of Grade A tertiary hospitals in Heilongjiang Province, may limit the generalisability of this study's findings.
2. The quality of the participants' self-recall or stigma and shame of the events of the past 12 months may result in an underestimation of the number of violent incidents.
3. Despite the limitations, this study can draw hospital managers' attention to the workplace violence that occurs in otorhinolaryngology departments, motivating them to reduce the occurrence of depressive symptoms in otorhinolaryngologists and endeavour to improve the quality of their medical service.

Funding

This study was funded by the Natural Science Foundation of China (Grant No. 71273002, 71473064，71673073); New Century Excellent Talents of University from the Ministry of Education, China (Grant No.1252-NCET02); the China Postdoctoral Science Foundation (2015M570211, 2016T90181); the Heilongjiang Provincial Association of Social Sciences (15058), and the Collaborative Innovation Centre of Social Risks Governance in Health.

For peer review only - http://bmjopen.bmj.com/site/about/guidelines.xhtml

Conflict of interest declaration: The authors declare no conflict of interest

Data sharing statement： Data are available from the corresponding author upon request.

For peer review only

For peer review only - http://bmjopen.bmj.com/site/about/guidelines.xhtml

1. INTRODUCTION

In October 2013,[1] a patient at the Zhejiang Provincial People's Hospital of Otorhinolaryngology in China stabbed to death the physician who was providing his diagnosis and treatment. Similarly, in February 2014, an otorhinolaryngologist at a hospital in Qiqihar City, Heilongjiang Province, China, was beaten to death while examining a patient. The frequency and severity of such workplace violence, (WPV), among medical professionals in China has increased in recent years, as has its public attention.[2] WPV refers to the physical and psychological harm that professionals may face while performing their duties.[3, 4] WPV is a significant increasing phenomenon in health care settings, and all health workers, especially nurses, are at risk of suffering aggressive assaults.[5] In fact, the number of WPV incidents reported in health-care facilities accounts for almost one-quarter of the total number of violent incidents

For peer review only - http://bmjopen.bmj.com/site/about/guidelines.xhtml

reported for all workplaces,[6] making healthcare workers the most vulnerable group to WPV.[7] Naturally, the occurrence of hospital violence may affect the health of physicians and nurses and hinder their professional performance, resulting in a negative impact on patient services and overall health.[8-10]

Instances of such violence can be found in several hospital departments,[11] and many studies have focused on the high incidence of violence in emergency rooms.[12, 13] However, we found a comparable rate of violent incidences in otorhinolaryngology departments. The China Hospital Association 2014 data show that from 2008 to 2012, incidences of severe hospital violence increased from 47.7% to 63.7% of all hospital violence [14] and, upon examining 30 cases of physicians and nurses who were victims of fatal WPV in China over the past 10 years, we determined that professionals working in otorhinolaryngology departments account for over 10% of the total.

The main causes of violence towards otorhinolaryngology physicians and nurses include (1) that patients tend to consider otorhinolaryngology-related diseases as less important; (2) that physicians and nurses in this department have limited professional skills compared to those in other departments; and (3) the treatment results of otorhinolaryngological diseases are not ideal, (i.e. many conditions cannot be immediately or completely cured).[15] Thus, considering these dangers, it is clear that the working environment of otorhinolaryngology physicians and nurses should be a concern for hospital administrations, both from a mental health and WPV perspective.

For peer review only - http://bmjopen.bmj.com/site/about/guidelines.xhtml

Previous studies have shown that WPV is a contributing factor to employees suffering from mental illnesses such as depression.[16-18] It is well-known that depression is a common illness, and it seems to be more prevalent in high-income countries; the incidence rate of depression among adults in low- to middle-income countries is 11.1%, compared to 14.6% in high-income countries[19, 20]; specifically, the incidences of depression in the United States, Canada, and the UK are 12%, 19.3%, and 15.5%, respectively.[21, 22] In Canada, one in 10 nurses have been found to have depressive symptoms,[23] and in France the situation is even more serious, with one-third of nurse managers suffering from such symptoms.[24] Depressive symptoms can negatively affect job satisfaction, work efficiency, and overall happiness, resulting in a negative attitude toward providing medical treatment. Over the long term, this can not only affect the development of human resources in health fields, but also reduce the quality of medical care provided.[9, 10, 25] Research shows that Chinese physicians and nurses experience various psychological issues, with the incidence of depression determined to be 34.2%.[26] In a study based in southern China, (specifically, Guangdong Province), depressive symptoms were found to be associated with WPV, long working hours, shift frequency, and departmental operations.[8] Also notable is the fact that studies have revealed a difference in the proportion of depressive symptoms among nurses in different hospital departments, which strongly suggests that managers should seek to determine the reason for these differences between departments through the use of their employee relations and other suitable processes.[8]

For peer review only - http://bmjopen.bmj.com/site/about/guidelines.xhtml

Further, it should also be noted that much of the existing research primarily assesses the overall situations of hospitals, with few studies focusing on specialised departments. Of the latter studies, the focus has primarily been placed on the emergency department; however, as mentioned above, the department of otolaryngology witnesses a similar number of violent phenomena as emergency rooms, and more than that found in other departments; therefore, the occurrence of depressive symptoms among otolaryngology staff should be taken seriously.

Otolaryngology is a key department in most hospitals, and has a number of unique characteristics when compared with other departments. First, there are many types of otolaryngology-related diseases, each presenting in unique anatomical locations, and these can affect patients' breathing, speech, smell, and hearing. Many such diseases have long cure times; therefore, providing patients with fast and effective treatment is not always possible. Second, the patient population of an otolaryngology department can span a wide range of age groups, resulting in relatively large workloads for physicians and nurses. Third, otolaryngology involves a variety of specialisations, requiring physicians and nurses to engage in continual learning to improve their professional and technological skills.[15, 27]

For these reasons, we hypothesise that the proportion of otolaryngology physicians and nurses suffering from depressive symptoms exceeds that of other hospital departments. While some scholars have studied the influencing factors depressive symptoms have on physicians and nurses in general, few existing studies

For peer review only - http://bmjopen.bmj.com/site/about/guidelines.xhtml

have assessed WPV-related factors associated with depressive symptoms among otorhinolaryngology physicians and nurses. Consequently, the purpose of this study is: (1) to investigate the prevalence of depressive symptoms among otorhinolaryngology physicians and nurses; and (2) to assess the risk factors that influence depressive symptoms, including WPV-related risk factors and demographic variables, and to present some specific suggestions.

2. METHODS

2.1. study population

We conducted a retrospective cross-sectional survey of otorhinolaryngologists and nurses from Grade A tertiary hospitals in Heilongjiang Province. Grade A tertiary hospitals are classified in accordance with China's current 'hospital classification management approach', which is designed to implement the division of medical institutions. When a medical institution has over 501 beds, it has the capability to provide high-level, specialised medical and health services in several areas, and also to implement higher-level education and scientific research tasks; such hospitals are ranked as 'tertiary'. Further, according to the scoring standard, those that score more than 900 points as general hospitals are categorised into the highest classification level for hospitals in mainland China.[28]

2.2 data collection

Considering the length of time allotted for this research and the limited time

For peer review only - http://bmjopen.bmj.com/site/about/guidelines.xhtml

available to clinical staff, we decided to adopt a web-based questionnaire approach, which would help to ensure the integrity and effectiveness of the data collection, and we also chose to apply a convenience sampling method. First, with the help of the chairman of the Heilongjiang Province branch of the Association of Otorhinolaryngology Physicians, we obtained approval from the Heilongjiang Provincial Association of Otorhinolaryngology to conduct the study. The association consequently provided a list of Grade A tertiary hospitals and associated physicians' email addresses, which allowed us to contact otolaryngology directors to seek assistance in acquiring related nurses' email addresses. We then sent these individuals an introductory email with a link to the informed consent form and questionnaire. After the initial e-mail was sent, a reminder e-mail was sent to any physicians and nurses who did not respond initially. Through this process, we obtained 652 valid questionnaires, (total efficiency of 83.6%). The data were collected from January 10, 2017, to February 15, 2017.

2.3. the questionnaire preparation

After obtaining written permission, we sent the respondents a questionnaire that was developed by the International Labor Office, (ILO), International Council of Nurses, (ICN), World Health Organization, (WHO), and Public Service International, (PSI) in 2003 with the goal of measuring violence in the workplace.[29] We translated the questionnaire into Mandarin and then back into English to verify the accuracy of the Mandarin version. We then invited the aforementioned experts identified by the

For peer review only

otorhinolaryngology association to modify the questions as needed, and then assessed the validity of the contents of the questionnaire, including its suitability for Chinese culture and appropriateness of the translation. These experts included epidemiologists, health-management specialists, public health specialists, health system specialists, otolaryngology experts, and administrative experts, (15 persons). We also conducted a two-week retest reliability check, (Cronbach's $\alpha = 0.87$), by having a group of 42 otorhinolaryngology physicians and nurses from five Grade A tertiary hospitals take the test, and then take it again two weeks later.

The final version of the questionnaire included four parts: (1) the demographic characteristics of the professionals, (gender, age, marital status, years of work experience, education level, occupation, seniority), and workplace-related conditions, number of patients received per day, shift work, number of co-workers for more than half of working hours, and anxiety experienced at work, (which was rated from 1, (absent), to 5, (extremely high),); (2) experience of physical violence in the previous 12 months, (defined as intentional injury to body); (3) experience of nonphysical violence over the previous 12 months, (including verbal abuse, threatening events, and sexual harassment); and (4) depressive symptoms, measured using a Chinese version of the Zung Self-Rating Depression Scale, (SDS), which has good reliability. SDS is usually used to measure the level of depressive symptoms in the general population over the previous week, and is widely used in research into WPV and depressive symptoms.[8, 30] Specifically, it facilitates the revealing of subjective feelings

For peer review only - http://bmjopen.bmj.com/site/about/guidelines.xhtml

using 20 items which are rated in terms of severity using a four-point scale ranging from 1, (absent or minimum), to 4, (high or extremely high). The total score was recorded as the original score, the scores for the 20 items were added together, and finally the resulting score was multiplied by 1.25 to get the standard score. A standard score of 53~62 points represents mild depression, 63~72 points represents moderate depression, and >72 points signifies severe depression. To ensure that the results of this study can be compared with the results of related studies, we chose to use the same classification method as other studies: setting a final score of ⩾ 53 points as indicating the presence of depressive symptoms.[31, 32]

2.4. data analysis

Descriptive statistical calculations were conducted on the respondents' demographic characteristics and their reported frequency of experiencing depressive symptoms; in addition, a chi-test was used to analyse the correlation between symptoms of depression and the respondents' characteristics, as well as WPV-related factors. Further, logistic regression analysis was used to determine the characteristics and factors, (including years of work experience, education level, seniority, number of patients received per day, number of co-workers for more than half of working hours, anxiety experienced at work, and physical violence), were likely to predict depression. SPSS v.19.0 was used to conduct the analysis, (statistical products and services solutions 19.0, 2010, IBM, Armonk, NY, USA). Statistical significance is indicated by $p < 0.05$.

For peer review only - http://bmjopen.bmj.com/site/about/guidelines.xhtml

2.5. ethics statement

All respondents received an informed consent form, which described the goal and method of the data collection, how the data would be handled, and also an assurance of confidentiality. All respondents who gave their informed consent completed the questionnaire. The study protocol was reviewed and approved by the Research Ethics Committee of Harbin Medical University.

3. RESULTS

In Table 1, the demographic characteristics of the study participants are shown. Of these otolaryngology staff, 63.7% were women, almost half, (41.7%), were between 30 and 39 years of age, and three-quarters, (78.1%), were married. The majority of respondents, (69.8%), had at least 10 years of experience in otolaryngology and 78.2% were graduated or specialised. Of the respondents, 19.5% was senior within the department.

Descriptive statistics concerning the depressive symptoms of the participants are presented in Table 2; notably, the table shows that 57.2% of the 652 otorhinolaryngology physicians and nurses had depressive symptoms. Moreover, approximately half. (49.54%). of the respondents worked with one or two colleagues for more than half of their working hours while, of the respondents with depression symptoms, three quarters. (76.41%). had experienced varying degrees of anxiety. Additionally, of the respondents who had suffered physical violence, 71.25% had

For peer review only - http://bmjopen.bmj.com/site/about/guidelines.xhtml

depressive symptoms. We then assessed associations between sample characteristics and depressive symptoms using Pearson chi-square tests and determined that years of work experience, seniority, number of co-workers for more than half of the working hours, anxiety experienced at work, and physical violence are significantly associated with depressive symptoms.

In Table 3, the variables related to depressive symptoms, determined using a multiple logistic model, an adjusted odds ratio, (OR), and a 95% confidence interval, (CI), are shown.Our otorhinolaryngology physicians and nurses who suffered from physical violence, (OR = 1.82, 95% CI = 1.06–3.12), were more likely to suffer from depressive symptoms than those who had not. Moreover, participants with less than one year of experience were also more likely to suffer from depressive symptoms, while those with more experience were progressively less likely to suffer from depressive symptoms. Otorhinolaryngology physicians and nurses who worked alone for more than half of their working hours were more likely to suffer from depressive symptoms than those who worked frequently with other colleagues. Relative to the seniority of otorhinolaryngology physicians and nurses, formal staff and those with junior and intermediate titles showed protective factors for depressive symptoms.

4. DISCUSSION

Our study examined the association between depressive symptoms and WPV-related risk factors in regard to otorhinolaryngology physicians and nurses in

For peer review only - http://bmjopen.bmj.com/site/about/guidelines.xhtml

northern China. Our results confirm that depressive symptoms are common among professionals in this department, with the incidence of depressive symptoms shown to be 57.2%. There is limited research on depression among otorhinolaryngology staff, so we could not directly compare our results with those of other studies; however, these results show a significantly higher prevalence of depressive symptoms than that found among general physicians and nurses in the United States, (11.3%),[33] US nurses (41%),[35] general physicians in the Netherlands, (29%),[34] and Iranian physicians and nurses, (28.76%).[36] Further, this number is also slightly higher than the incidence of depressive symptoms reported among emergency department nurses in Taiwan.[1] In addition, the studies of Yang and Ji et al. reported that the incidence of depressive symptoms among healthcare workers in China across all hospital departments is 45.3% and 47.2%, respectively[37, 38]; however, the incidence of depression differs between departments. Compared with other departments, otorhinolaryngology physicians and nurses have a higher incidence of depressive symptoms than most other departments outside of emergency, and also report higher than normal levels[8, 30]; however, it should be noted that few previous studies have focused on the incidence of depressive symptoms among these professionals in China. Unfortunately, in China, the frequency of violence (which is a major predictor of depression) within otorhinolaryngology department is relatively high due to various factors involving treatment options and professional skill levels[15]; therefore, research into the association between depressive symptoms and WPV-related risk factors is essential for preserving not only the mental

For peer review only - http://bmjopen.bmj.com/site/about/guidelines.xhtml

health of practitioners, but also for ensuring quality of care.

This study found several factors associated with the occurrence of depressive symptoms. First, years of work experience and seniority were identified; the fewer a physician's or nurse's years of work experience, the more likely they are to suffer from depressive symptoms. This has also been confirmed in previous studies on healthcare professionals in China.[39] Less-experienced physicians and nurses often receive insufficient social support, and are more likely be affected by occupational stress.[39, 40] Thus, the mental state of physicians and nurses with few years of experience should be evaluated as early as possible in order to provide them with preventative and proactive psychological support, as well as training opportunities.[41]

Second, considering WPV-related risk factors, number of co-workers for more than half of working hours was found to be a predictor of depression among otorhinolaryngology professionals. The results of the survey showed that, when compared with physicians and nurses who mainly work with two colleagues, those who frequently work alone are more likely to suffer from depressive symptoms; the higher the number of colleagues working together, the lower the risk of depressive symptoms. Working alongside colleagues can help physicians and nurses handle pressure, dissatisfaction, and other emotional fluctuations. Previous studies have also shown that social support can reduce the incidence of depressive symptoms, help individuals manage stress, and promote good mental health.[42] Peer support intervention, in particular, can effectively reduce depressive symptoms, not only

For peer review only - http://bmjopen.bmj.com/site/about/guidelines.xhtml

because it can be implemented through daily interactions, but also because it can have the same effect as group cognitive behavioural therapy.[43] Support at work is a social-network resource associated with the work environment, and employees at all levels can benefit from the support of their colleagues. In the demand-control-support model, it has been highlighted that social support is similar to job control, and may have a buffering or reinforcing effect on individuals' psychological responses; for example, work friendship between colleagues has been reported to be an important social support.[44] Therefore, the working status of medical staff, (e.g. time spent working alone), should be considered in order to strengthen peer support, (as well as team cooperation), and thereby effectively prevent and improve depressive symptoms.

Third, this study revealed that physical violence is an important predictor of incidences of depressive symptoms within the otolaryngology department. Our findings suggest that the risk of physicians and nurses who have suffered physical violence developing depressive symptoms is 1.82 times greater than that for professionals who have not experienced such violence. This is supported by previous surveys, which have also indicated that WPV is a strong predictor of psychological problems such as depressive symptoms.[16] Depression is one of several negative yet common consequences experienced by physicians and nurses who suffer from WPV.[45]Among them, physical violence is more likely to cause physicians and nurses to experience depressive symptoms.[39] The reasons for the high rate of physical violence within otolaryngology departments are varied, but an important factor is that

For peer review only - http://bmjopen.bmj.com/site/about/guidelines.xhtml

otorhinolaryngology-related diseases are often complex and involve longer courses of treatment, which can lead to patient insomnia, anxiety, and other psychological problems, which can in turn lead to a loss of trust in physicians and nurses. As such, these patients can be prone to adverse emotions.[46] Further, due to the nature of many otorhinolaryngology diseases, which affect speech and hearing, patients are often unable to effectively express their dissatisfaction, making them more inclined to choose physical violence to express their discomfort or psychological distress.[47]

Physical violence can also cause psychological damage to physicians and nurses; for example, it can increase levels of anxiety, which can result in poor productivity, lower quality of work, a decline in individual sense of accomplishment, and poor relationships with patients, managers, and colleagues.[12, 47, 48] In addition, physical violence can also have a negative impact on physicians and nurses' social relationships both inside and outside the hospital.[48] Consequently, the relationship between physicians and nurses and their patients should be improved through different measures, including by attaching importance to patients' psychological status during clinical diagnosis and treatment processes, enhancing the communication skills of otorhinolaryngology staff, and improving patients' awareness of their disease and potential treatment options, (including duration and adverse side-effects). Concurrently, medical institutions should adopt a 'zero tolerance' approach to violent incidents and, to reduce the harm caused by physical violence, managers should strengthen the provision of timely psychological comfort and psychological

For peer review only - http://bmjopen.bmj.com/site/about/guidelines.xhtml

counselling for employees who have experienced physical violence.

In previous studies, the incidence of depressive symptoms was associated with physicians and nurses' workload and shift characteristics[49-53]; however, this study did not find any relevant association between depression and these two factors among otorhinolaryngology professionals, similar to the findings of another study of resident physicians in Turkey.[54] Future research should further explore this area.

This study has the following limitations. First, we only investigated otorhinolaryngology physicians and nurses working in Grade A tertiary hospitals in Heilongjiang Province, so the results may not be fully representative of the entire situation in northern China, and cannot be extended to other types of hospitals or departments. Future studies should conduct in-depth exploration of the different types of otorhinolaryngological services at hospitals of different grades. Second, physicians and nurses who were absent from work because of depression were not assessed; thus, the reported rate of depressive symptoms may be lower than the true value. Third, because the respondents were asked to review the events of the past 12 months, the prevalence of violence could have been under-reported as a result of reporting or recall bias or stigma and shame. Fourth, this study focuses on the more distinctive WPV-related factors for this analysis; however, there may be additional factors related to the occurrence of depressive symptoms that could be further explored. Nevertheless, the results of this study indicate that physical violence and mainly working alone are risk factors for depressive symptoms among otorhinolaryngology physicians and

For peer review only - http://bmjopen.bmj.com/site/about/guidelines.xhtml

nurses, and this provides some basis and direction for future research.

In conclusion, the results show that otorhinolaryngology physicians and nurses in Grade A tertiary hospitals in Heilongjiang Province, China, have a high risk of experiencing depressive symptoms. Of the 652 respondents, 57.2% reported varying degrees of depressive symptoms. Our findings also show that experiencing physical violence, number of co-workers for more than half of working hours, and other WPV-related factors are associated with symptoms of depression. Therefore, on one hand, it is clear that medical institutions should pay more attention to otorhinolaryngology. Essential measures include improving communication between medical staff and patients and the adoption of a 'zero tolerance' attitude towards hospital violence. On the other hand, attention should be paid to the construction of team relationships, improving teams' cooperation, and giving full backing to peer support to alleviate depressive symptoms. Finally, more attention should also be paid to lateral violence, (bullying, harassment, physical violence between staff, etc.) to address depressive symptoms caused by such violence. However, as we did not study the impact of lateral violence on depressive symptoms, this represents an avenue of research for future studies.

References

1. Hsieh, H.F., et al., Association among components of resilience and workplace violence - related depression among emergency department nurses in Taiwan: a cross - sectional study. Journal of Clinical Nursing, 2016. **25**(17-18): p.

For peer review only - http://bmjopen.bmj.com/site/about/guidelines.xhtml

2639-2647.

2. Qiang Ma, An Analysis of the Causes of Frequent Cases of Violent Injuries. National Medical Journal of China, 2014. 94(18): p. 1368-1370.

3. Healthcarefacilities, et al., Violence - Occupational hazards in hospitals. Hospitals, 2002.

4. Simpson, K.R., Incivility, Bullying, and Workplace Violence. Mcn the American Journal of Maternal/child Nursing, 2016. **41**(1): p. 68.

5. Paola, F., et al., Workplace violence in different settings and among various health professionals in an Italian general hospital: a cross-sectional study. Psychology Research & Behavior Management, 2016. **9**: p. 263-275.

6. ISBN, Framework guidelines for addressing workplace violence in the health sector. Geneva Switzerland Ilo, 2002.

7. Sun, P., et al., Workplace Violence against Health Care Workers in North Chinese Hospitals: A Cross-Sectional Survey. International Journal of Environmental Research & Public Health, 2017. **14**(1).

8. Gong, Y., et al., Prevalence of depressive symptoms and work-related risk factors among nurses in public hospitals in southern China: A cross-sectional study. Scientific Reports, 2014. **4**: p. 7109.

9. Landrigan, C.P., et al., Effect of reducing interns' work hours on serious medical errors in intensive care units. New England Journal of Medicine, 2004. **351**(18): p. 1838-1848.

10. Lockley, S.W., et al., Effect of reducing interns' weekly work hours on sleep and attentional failures. New England Journal of Medicine, 2004. **351**(18): p. 1829.

For peer review only - http://bmjopen.bmj.com/site/about/guidelines.xhtml

11. Yan, T., et al., Perceived Risk of Violence in Various Hospital Levels and Departments in Urban and Rural China. Iranian Journal of Public Health, 2016. **45**(9): p. 1238-1240.

12. Eslamian, J., A.A. Akbarpoor, and S.A. Hoseini, Quality of work life and its association with workplace violence of the nurses in emergency departments. Iranian Journal of Nursing & Midwifery Research, 2015. **20**(1): p. 56-62.

13. Stene, J., et al., Workplace violence in the emergency department: giving staff the tools and support to report. Permanente Journal, 2015. **19**(2): p. e113-7.

14. Lingling Wang, Chen Wang, Yanlin Cao, Yue Zhao, Yutao Cheng, Lili Zheng., Analysis and thoughts on trend and harm of hospital violence. Chinese Hospitals., 2014(3): p. 4-6.

15. Kai Zhang and Kang Xue, Difficulties and Countermeasures of Young Doctors in the Department of ENT in Primary Hospitals. Medicine and Law, 2016. 8(4): p. 45-47.

16. Aytaç, S. and S. Dursun, The effect on employees of violence climate in the workplace. Work, 2012. **41 Suppl 1**(6): p. 3026.

17. Merecz, D., M. Drabek, and A. Mościcka, Aggression at the workplace--psychological consequences of abusive encounter with coworkers and clients. International Journal of Occupational Medicine & Environmental Health, 2009. **22**(3): p. 243.

18. Silva, A.T.C.D., et al., Violence at work and depressive symptoms in primary health care teams: a cross-sectional study in Brazil. Social Psychiatry and Psychiatric Epidemiology, 2015. **50**(9): p. 1-9.

19. Brundtland, G.H., Mental Health: New Understanding, New Hope. Journal of the American Medical Association, 2001. **286**(19): p. 2391-2391.

For peer review only

20. Bromet, E., et al., Cross-national epidemiology of DSM-IV major depressive episode. BMC Medicine, 2011. **9**(1): p. 90-90.

21. Coomber, S., et al., Stress in UK intensive care unit doctors. Bja British Journal of Anaesthesia, 2002. **89**(6): p. 873.

22. Lloyd, S., D. Streiner, and S. Shannon, Burnout, depression, life and job satisfaction among Canadian emergency physicians. Journal of Emergency Medicine, 1994. **12**(4): p. 559-565.

23. Ohler, M.C., M.S. Kerr, and D.A. Forbes, Depression in nurses. The Canadian journal of nursing research = Revue canadienne de recherche en sciences infirmières, 2010. **42**(3): p. 66-82.

24. Nourry, N., et al., Psychosocial and organizational work environment of nurse managers and self-reported depressive symptoms: cross-sectional analysis from a cohort of nurse managers. International Journal of Occupational Medicine and Environmental Health, 2014. **27**(2): p. 252-69.

25. Xiaoying Jiang, Ji Gao, and Xuan Zhang, Correlation analysis about workplace violence and job burnout among the head nurses. Chinese Nursing Management, 2008. 8(3): p. 18-20.

26. Yanqin Yuan, Bo Wang, Xianni Wang, Kuihua Jin., Survey on Mental Health Status of the Medical Staff. Nursing Journal of Chinese People's Liberation Army, 2007. 24(12): p. 22-23.

27. Wei Li, Analysis of Psychological Characteristics of Patients with Otorhinolaryngology and Analysis of Psychological Nursing, Modern Diagnosis & Treatment, 2014(2): p. 1428-1429.

28. Yan Li. Interpretation and preparation of the evaluation standard of tertiary general hospital (2011 Edition) by the Ministry of health. Preparation of [C]//

For peer review only - http://bmjopen.bmj.com/site/about/guidelines.xhtml

"hospital accreditation standards and assessment management interpretation" training paper. 2011.

29. ILO/ICN/WHO/PSI, Workplace Violence in the Health Sector Country Case Stusy-Questionnaire. 2003: p. 1-14.

30. Gong, Y., et al., Prevalence of Anxiety and Depressive Symptoms and Related Risk Factors among Physicians in China: A Cross-Sectional Study. Plos One, 2014. **9**(7): p. e103242.

31. Zhengyu Wang and Yufen Chi, Self-Rating Depression(SDS). Shanghai Archives of Psychiatry, 1984(2).

32. Zung, W.W.K., A Self-Rating Depression Scale. Archives of General Psychiatry, 1965. **12**(12): p. 63-70.

33. Schwenk, T.L., D.W. Gorenflo, and L.M. Leja, A survey on the impact of being depressed on the professional status and mental health care of physicians. Journal of Clinical Psychiatry, 2008. **69**(4): p. 617-20.

34. Ruitenburg, M.M., H.W. Frings-Dresen Monique, and J.K. Sluiter, The prevalence of common mental disorders among hospital physicians and their association with self-reported work ability: a cross-sectional study. BMC Health Services Research, 2012. **12**(1): p. 1-7.

35. Ruggiero, J.S., Health, work variables, and job satisfaction among nurses. Journal of Nursing Administration, 2005. **35**(5): p. 254-263.

36. Roohafza, H.R., et al., What's the role of perceived social support and coping styles in depression and anxiety? Journal of Research in Medical Sciences the Official Journal of Isfahan University of Medical Sciences, 2014. **19**(10): p. 944-9.

37. Xiaofang Yang and Shaoyun Hu, Related Study between Social Support and

For peer review only

Coping Style, and Anxiety and Depression of Nurse. China Journal of Health Psychology, 2011. 19(3): p. 300-301.

38. Yongjuan Ji, Lina He, Jing Hao, Guifang Kuang., Correlation study of occupational stress and mental health among medical staff, Occupation and Health, 2015. 31(24): p. 3393-3395.

39. Lijia Tian, Occupational stress and its relationship to depressive symptoms and anxiety disotder among ophthalmologists in Shanghai.2013, Fudan University.

40. Raffaelli, M., et al., Stress, Social Support, and Depression: A Test of the Stress-Buffering Hypothesis in a Mexican Sample. Journal of Research on Adolescence, 2013. **23**(2): p. 283–289.

41. Hassan, T.M., et al., A postal survey of doctors' attitudes to becoming mentally ill. 2009. **9**(4): p. 327-332.

42. Hsieh, H.F., et al., Association among components of resilience and workplace violence-related depression among emergency department nurses in Taiwan: a cross-sectional study. Journal of Clinical Nursing, 2016. **25**(17-18): p. 2639-2647.

43. Piette, J.D., Efficacy of peer support interventions for depression: a meta-analysis. General Hospital Psychiatry, 2011. **33**(1): p. 29.

44. Qian Shi, Xiaobing Shu, and Yuyue Luo, The job demand-control-support model a review of empirical research. Advances in Psychological Science, 2010. **18**(4): p. 655-663.

45. Alexy, E.M. and J.A. Hutchins, Workplace violence: a primer for critical care nurses. Critical Care Nursing Clinics of North America, 2006. **18**(3): p. 305-12.

For peer review only

For peer review only - http://bmjopen.bmj.com/site/about/guidelines.xhtml

46. Yu Liu and Weiping He, Ethical Reflections on Medical Dispute in the Department of Otorhinolaryngology. Chinese Medical Ethics, 2016. 29(5): p. 777-779.

47. Roldán, G.M., et al., Violence at work and its relationship with burnout, depression and anxiety in healthcare professionals of the emergency services. Health, 2013. **05**(2): p. 193-199.

48. Y, D., et al., Bullying among nurses and its effects. International Nursing Review, 2009. **56**(4): p. 504-511.

49. Lee, H.E., H.R. Kim, and J.S. Park, Work-related risk factors for workplace violence among Korean employees. Journal of Occupational Health, 2014. **56**(1): p. 12.

50. Virtanen, M. and M. Kivimäki, Saved by the bell: does working too much increase the likelihood of depression? Expert Review of Neurotherapeutics, 2012. **12**(5): p. 497-499.

51. Bannai, A. and A. Tamakoshi, The association between long working hours and health: a systematic review of epidemiological evidence. Scandinavian Journal of Work Environment & Health, 2014. **40**(1): p. 5-18.

52. Oliveira, F.P.D., et al., Symptoms of depression and intervening factors among nurses of emergency hospital services. Acta Paulista De Enfermagem, 2015. **28**(3): p. 209-215.

53. Paola, F., et al., The impact of shift work on the psychological and physical health of nurses in a general hospital: a comparison between rotating night shifts and day shifts. Risk Management & Healthcare Policy, 2016. **9**: p. 203.

54. Demir, F., et al., [The prevalence of depression and its associated factors among resident doctors working in a training hospital in Istanbul]. Türk

For peer review only

psikiyatri dergisi = Turkish journal of psychiatry, 2007. **18**(1): p. 31.

Author contributions

H.F., X.Z., H.Y. drafted the manuscript; J.M. designed the study; P.S., Y.L., K.J., and P.L. collected the data; J.M., M.L., H.Q., and Q.W. analyzed the data; J.M. contributed to the manuscript's revision. All authors approved the final manuscript for publication.

For peer review only

Table 1. Participant demographic characteristics (N = 652).

Characteristic	n	%
**Gender**		
Male	237	36.3%
Female	415	63.7%
**Age**		

For peer review only - http://bmjopen.bmj.com/site/about/guidelines.xhtml

≤29	214	32.8%
30–39	272	41.7%
≥40	166	25.5%
**Marital status**		
Unmarried	130	19.9%
Married	509	78.1%
Divorced	9	1.4%
Widowed	1	0.2%
Other	3	0.5%
**Years of work experience**		
<1	76	11.7%
1-10	379	58.1%
11-20	126	19.3%
21-30	61	9.4%
>30	10	1.5%
**Seniority**		

For peer review only
For peer review only - http://bmjopen.bmj.com/site/about/guidelines.xhtml

Senior	127	19.5%
Intermediate	237	36.3%
Primary	168	25.8%
No title (formal doctor)	65	10.0%
Interns (resident training in hospital or post-graduate)	55	8.4%
**Education level**		
Graduate and above	130	19.9%
Undergraduate	380	58.3%
Community college	97	14.9%
Vocational school	40	6.1%
Other	5	0.8%
**Occupation**		
Physician	379	58.1%
Nurse	273	41.9%

For peer review only - http://bmjopen.bmj.com/site/about/guidelines.xhtml

Table 2. Descriptive statistics associated with depressive symptoms of the participants.

**Variables**	**Non-depressive tendency N = 279(42.8%) n (%)**	**Depressive tendency N = 373 (57.2%) n (%)**	x^2	**P**
**Gender**			0.01	0.92
Male	102 (36.56%)	135 (36.19%)		
Female	177 (63.44%)	238 (63.81%)		
**Age**				
≤29	94 (33.69%)	120 (32.17%)	0.20	0.91
30–39	114 (40.86%)	158 (42.36%)		
≥40	71 (25.45%)	95 (25.47%)		
**Marital status**			4.92	0.30
Unmarried	49 (17.56%)	81 (21.72%)		
Married	226 (81.00%)	283 (75.88%)		
Divorced	2 (0.72%)	7 (1.88%)		

For peer review only - http://bmjopen.bmj.com/site/about/guidelines.xhtml

Widowed	0 (0%)	1 (0.27%)		
Other	2 (0.72%)	1 (0.27%)		
**Years of work experience**			10.26	0.04
<1	33 (11.83%)	43 (11.53%)		
1-10	163 (58.42%)	216 (57.91%)		
11-20	48 (17.20%)	78 (20.91%)		
21-30	26 (9.32%)	35 (9.38%)		
>30	9 (3.23%)	1 (0.27%)		
**Seniority**			14.00	0.01
Senior	52 (18.64%)	75 (20.11%)		
Intermediate	89 (31.90%)	148 (39.68%)		
Primary	69 (24.73%)	99 (26.54%)		
No title (formal doctor)	39 (13.98%)	26 (6.97%)		
Interns (resident training in hospital or post-graduate)	30 (10.75%)	25 (6.70%)		

For peer review only

**Education level**			7.32	0.12
Graduate and above	52 (18.64%)	78 (20.91%)		
Undergraduate	174 (62.37%)	206 (55.23%)		
Community college	31 (11.11%)	66 (17.69%)		
Vocational school	20 (7.17%)	20 (5.36%)		
Others	2 (0.72%)	3 (0.80%)		
**Occupation**			0.45	0.50
Physicians	158(56.63%)	221(59.25%)		
Nurses	121(43.37%)	152(40.75%)		
**Number of patients received per day**			1.02	0.91
1-20	122 (43.73%)	154 (41.29%)		
21-30	83 (29.75%)	116 (31.10%)		
31-40	39 (13.98%)	55 (14.75%)		
41-50	13 (4.66%)	22 (5.90%)		
>51	22 (7.89%)	26 (6.97%)		

For peer review only

For peer review only - http://bmjopen.bmj.com/site/about/guidelines.xhtml

**Shift work**			0.01	0.92
Yes	222 (79.57%)	298 (79.89%)		
No	57 (20.43%)	57 (15.28%)		
**Number of coworkers for more than half of the working hours**			13.62	0.01
1	17 (6.09%)	40 (10.72%)		
2	107 (38.35%)	159 (42.63%)		
3-5	97 (34.77%)	129 (34.58%)		
6-10	35 (12.54%)	32 (8.58%)		
>10	23 (8.24%)	13 (3.49%)		
**Anxiety experienced at work**			9.54	0.04
Absent	89 (31.90%)	88 (23.59%)		
Low	52 (18.64%)	61 (16.35%)		
Moderate	74 (26.52%)	105 (28.15%)		
High	23 (8.24%)	38 (10.19%)		

For peer review only

Extremely high	41 (14.70%)	81 (21.72%)		
**Physical Violence**			3.34	0.01
Yes	23 (8.24%)	57 (15.28%)		
No	256 (91.76%)	316 (84.72%)		
**Non-Physical Violence**			3.43	0.06
Yes	91 (32.62%)	148 (39.68%)		
No	188 (67.38%)	225 (60.32%)		

For peer review only

For peer review only - http://bmjopen.bmj.com/site/about/guidelines.xhtml

Table 3. Multiple logistic regression of depressive and non-depressive symptoms.

	OR	95% CI	P
**Years of work experience**			
<1	1.00	Reference	
1-10	0.04	0.01–0.39	0.01
11-20	0.06	0.01–0.52	0.01
21-30	0.06	0.01–0.52	0.01
>30	0.07	0.01–0.66	0.02
**Education level**			
Graduate and above	1.00	Reference	
Undergraduate	1.32	0.20–8.89	0.78
Community college	1.38	0.21–8.85	0.74
Vocational school	0.67	0.10–4.46	0.68
Others	1.45	0.21–10.13	0.71
**Seniority**			

For peer review only - http://bmjopen.bmj.com/site/about/guidelines.xhtml

Senior	1.00	Reference	
Intermediate	0.37	0.16–0.85	0.02
Primary	0.35	0.17–0.73	0.01
No title (formal doctor)	0.48	0.23–0.99	0.04
Interns (resident training in hospital or post-graduate)	0.92	0.40–2.15	0.85
**Number of patients received per day**			
1-20	1.00	Reference	
21-30	1.03	0.52–2.06	0.93
31-40	0.98	0.48–1.98	0.94
41-50	0.88	0.40–1.93	0.75
>51	0.71	0.27–1.87	0.49
**Number of coworkers for more than half of the**			

For peer review only
For peer review only - http://bmjopen.bmj.com/site/about/guidelines.xhtml

**working hours**			
1	1.00	Reference	
2	0.27	0.11–0.70	0.01
3-5	0.41	0.20–0.89	0.02
6-10	0.41	0.19–0.91	0.03
>10	0.73	0.30–1.74	0.47
**Anxiety experienced at work**			
Absent	1.00	Reference	
Low	1.84	1.09–3.09	0.02
Moderate	1.61	0.91–2.83	0.10
High	1.30	0.78–2.15	0.31
Extremely high	1.13	0.58–2.21	0.72
**Physical Violence**			
Yes	1.82	1.06–3.12	0.03
No	1.00	Reference	

For peer review only

For peer review only - http://bmjopen.bmj.com/site/about/guidelines.xhtml

STROBE 2007 (v4) Statement—Checklist of items that should be included in reports of *cross-sectional studies*

Section/Topic	Item #	Recommendation	Reported on page #
**Title and abstract**	1	(*a*) Indicate the study's design with a commonly used term in the title or the abstract	1
		(*b*) Provide in the abstract an informative and balanced summary of what was done and what was found	3-4
**Introduction**			
Background/rationale	2	Explain the scientific background and rationale for the investigation being reported	6-8
Objectives	3	State specific objectives, including any prespecified hypotheses	8-9
**Methods**			
Study design	4	Present key elements of study design early in the paper	9
Setting	5	Describe the setting, locations, and relevant dates, including periods of recruitment, exposure, follow-up, and data collection	9-13
Participants	6	(*a*) Give the eligibility criteria, and the sources and methods of selection of participants	9-10
Variables	7	Clearly define all outcomes, exposures, predictors, potential confounders, and effect modifiers. Give diagnostic criteria, if applicable	10-12
Data sources/ measurement	8*	For each variable of interest, give sources of data and details of methods of assessment (measurement). Describe comparability of assessment methods if there is more than one group	10-12
Bias	9	Describe any efforts to address potential sources of bias	*10*

For peer review only

Study size	10	Explain how the study size was arrived at	10
Quantitative variables	11	Explain how quantitative variables were handled in the analyses. If applicable, describe which groupings were chosen and why	10-12
Statistical methods	12	(*a*) Describe all statistical methods, including those used to control for confounding	12-13
		(*b*) Describe any methods used to examine subgroups and interactions	12-13
		(*c*) Explain how missing data were addressed	10
		(*d*) If applicable, describe analytical methods taking account of sampling strategy	13
		(*e*) Describe any sensitivity analyses	
**Results**			
Participants	13*	(a) Report numbers of individuals at each stage of study—eg numbers potentially eligible, examined for eligibility, confirmed eligible, included in the study, completing follow-up, and analysed	10
		(b) Give reasons for non-participation at each stage	
		(c) Consider use of a flow diagram	
Descriptive data	14*	(a) Give characteristics of study participants (eg demographic, clinical, social) and information on exposures and potential confounders	13/26-27
		(b) Indicate number of participants with missing data for each variable of interest	13
Outcome data	15*	Report numbers of outcome events or summary measures	14
Main results	16	(*a*) Give unadjusted estimates and, if applicable, confounder-adjusted estimates and their precision (eg, 95% confidence interval). Make clear which confounders were adjusted for and why they were included	14/28-34
		(*b*) Report category boundaries when continuous variables were categorized	28-34

For peer review only

		(*c*) If relevant, consider translating estimates of relative risk into absolute risk for a meaningful time period	
Other analyses	17	Report other analyses done—eg analyses of subgroups and interactions, and sensitivity analyses	
**Discussion**			
Key results	18	Summarise key results with reference to study objectives	14-15
Limitations	19	Discuss limitations of the study, taking into account sources of potential bias or imprecision. Discuss both direction and magnitude of any potential bias	19
Interpretation	20	Give a cautious overall interpretation of results considering objectives, limitations, multiplicity of analyses, results from similar studies, and other relevant evidence	15-19
Generalisability	21	Discuss the generalisability (external validity) of the study results	19-20
**Other information**			
Funding	22	Give the source of funding and the role of the funders for the present study and, if applicable, for the original study on which the present article is based	4-5

*Give information separately for cases and controls in case-control studies and, if applicable, for exposed and unexposed groups in cohort and cross-sectional studies.

Note: An Explanation and Elaboration article discusses each checklist item and gives methodological background and published examples of transparent reporting. The STROBE checklist is best used in conjunction with this article (freely available on the Web sites of PLoS Medicine at http://www.plosmedicine.org/, Annals of Internal Medicine at http://www.annals.org/, and Epidemiology at http://www.epidem.com/). Information on the STROBE Initiative is available at www.strobe-statement.org.

For peer review only

BMJ Open

Depressive symptoms and workplace violence-related risk factors among otorhinolaryngology nurses and physicians in northern China: A cross-sectional study

Journal:	*BMJ Open*
Manuscript ID	bmjopen-2017-019514.R2
Article Type:	Research
Date Submitted by the Author:	15-Dec-2017
Complete List of Authors:	Fang, Huiying; Harbin Medical University, Department of Health, Policy and Hospital Management, School of Public Health<br>Zhao, Xiao; Harbin Medical University School of Public Health, Department of Health Economy<br>Yang, Hai; the 2nd Affiliated Hospital of Harbin Medical University, Neurosurgery<br>Sun, Peihang; Harbin Medical University, Policy and Hospital Management<br>Li, Ying; Harbin Medical University, Policy and Hospital Management<br>Jiang, Ke; Harbin Medical University School of Public Health, Policy and Hospital Management<br>peng, li<br>Jiao, Mingli; Harbin Medical University, Health policy<br>Liu, Ming; the 2nd Affiliated Hospital of Harbin Medical University, Otorhinolaryngology<br>Qiao, Hong; the 2nd Affiliated Hospital of Harbin Medical University, Endocrine and Metabolic Diseases<br>Wu, Qunhong; Harbin Medical School, Social Medicine
**Primary Subject Heading**:	Public health
Secondary Subject Heading:	Ear, nose and throat/otolaryngology, Health policy
Keywords:	workplace violence, otorhinolaryngology, depressive symptoms, workplace violence-related risk factors.

SCHOLARONE™
Manuscripts

Depressive symptoms and workplace violence-related risk factors among otorhinolaryngology nurses and physicians in northern China: A cross-sectional study

Huiying Fang[1 †], Xiaowen Zhao[2 †], Haicheng Yang[3 †], Peihang Sun[4], Ying Li[5], Kexin Jiang[6], Peng Li[7], Mingli Jiao[8 *], Ming Liu[9 *], Hong Qiao[10 *], Qunhong Wu[11 *]

[1] Department of Health. Policy and Hospital Management, School of Public Health, Harbin Medical University, Harbin 150081, China; Email: 565453428@qq.com

[2] Department of Health Economy, School of Public Health, Harbin Medical University, Harbin 150081, China; Email: zhaoxiaowen12345@126.com

[3] Neurosurgery, the 2nd Affiliated Hospital of Harbin Medical University, Harbin 150081, China; Email: yanghaicheng123456@163.com

[4] Department of Health. Policy and Hospital Management, School of Public Health, Harbin Medical University, Harbin 150081, China; Email: 745450570@qq.com

[5] Department of Health. Policy and Hospital Management, School of Public Health, Harbin Medical University, Harbin 150081, China; Email: 1690342267@qq.com

[6] Department of Health. Policy and Hospital Management, School of Public Health, Harbin Medical University, Harbin 150081, China; Email: 1617358621@qq.com

[7] Department of Health. Policy and Hospital Management, School of Public Health, Harbin Medical University, Harbin 150081, China; Email: 656553133@qq.com

[8] Institute of Quantitative and Technical Economics, Chinese Academy of Social Science, Beijing 100000, China; Department of Health. Policy and Hospital

For peer review only - http://bmjopen.bmj.com/site/about/guidelines.xhtml

Management, School of Public Health, Harbin Medical University, Harbin 150081; Email: minglijiao@126.com

[9] Otorhinolaryngology, the 2nd Affiliated Hospital of Harbin Medical University, Harbin 150081, China; liuming6293@126.com

[10] Endocrine and Metabolic Diseases, the 2nd Affiliated Hospital of Harbin Medical University, Harbin 150081, China; qiaoh0823@sina.com

[11] Department of Social Medicine, School of Public Health, Harbin Medical University, Harbin 150081, China; qunhongwu@126.com

†These authors contributed equally to this work.

*Corresponding author: minglijiao@126.com (M.J.); liuming6293@126.com (M.L.); qiaoh0823@sina.com (H.Q.); qunhongwu@126.com (Q.W.); Tel.: +86-136-1367-5693 (M.J.); +86-131-0087-6901 (M.L.); +86-133-5986-4888 (H.Q.); +86-451-8750-2851 (Q.W.)

Word count: 3,691

For peer review only

Abstract

Objectives: Workplace violence is relatively frequent among medical professionals who work in otorhinolaryngology units. This phenomenon not only reduces the quality of provided medical care, but also increases the incidence of depressive symptoms among physicians and nurses, seriously affecting their job satisfaction and work efficiency with a negative attitude toward providing treatment. Few existing studies have assessed workplace-violence-related factors associated with depressive symptoms among otorhinolaryngology physicians and nurses.

Methods: We conducted a cross-sectional study in Grade A tertiary hospitals of Heilongjiang Province in northern China, to evaluate the occurrence and level of depressive symptoms among otorhinolaryngology physicians and nurses and to analyse the relationship between them and workplace-violence-related risk factors and demographic variables.

Results: Of all our participating professionals, (379 otorhinolaryngologists and 273 nurses), 57.2% was found to have depressive symptoms, whereas, of the respondents who had suffered from physical violence, 71.25% had depressive symptoms. Professionals with less than one year of experience, as well as professionals who more frequently worked alone, were more likely to suffer from depressive symptoms than their colleagues.

Conclusions: This research addresses an emerging issue of clinical practice, and its results differ from those of previous studies; specifically, it indicates that the frequency of depressive symptoms among otorhinolaryngology physicians and nurses

For peer review only - http://bmjopen.bmj.com/site/about/guidelines.xhtml

may be influenced by physical violence, the number of co-workers they have for more than half of their working hours, and other workplace-violence-related factors. To reduce the depressive symptoms caused by workplace violence and improve the quality of medical services, medical institutions should implement effective measures to prevent the occurrence of physical violence, strengthen team cooperation ability, and increase peer support.

Keywords: Workplace violence; otorhinolaryngology; depressive symptoms; workplace violence-related risk factors.

STRENGHTS AND LIMITATIONS OF THIS STUDY

1. The sample size, which is based on the number of Grade A tertiary hospitals in Heilongjiang Province, may limit the generalisability of this study's findings.
2. The quality of the participants' self-recall or stigma and shame of the events of the past 12 months may result in an underestimation of the number of violent incidents.
3. Despite the limitations, this study can draw hospital managers' attention to the workplace violence that occurs in otorhinolaryngology departments, motivating them to reduce the occurrence of depressive symptoms in otorhinolaryngologists and endeavour to improve the quality of their medical service.

Funding

This study was funded by the Natural Science Foundation of China (Grant No. 71273002, 71473064，71673073); New Century Excellent Talents of University from

For peer review only - http://bmjopen.bmj.com/site/about/guidelines.xhtml

the Ministry of Education, China (Grant No.1252-NCET02); the China Postdoctoral Science Foundation (2015M570211, 2016T90181); the Heilongjiang Provincial Association of Social Sciences (15058), and the Collaborative Innovation Centre of Social Risks Governance in Health.

Conflict of interest declaration: The authors declare no conflict of interest

Data sharing statement： Data are available from the corresponding author upon request.

For peer review only

1. INTRODUCTION

In October 2013,[1] a patient at the Zhejiang Provincial People's Hospital of Otorhinolaryngology in China stabbed to death the physician who was providing his diagnosis and treatment. Similarly, in February 2014, an otorhinolaryngologist at a hospital in Qiqihar City, Heilongjiang Province, China, was beaten to death while examining a patient. The frequency and severity of such workplace violence, (WPV), among medical professionals in China has increased in recent years, as has its public attention.[2] WPV refers to the physical and psychological harm that professionals may face while performing their duties.[3, 4] WPV is a significant increasing phenomenon in health care settings, and all health workers, especially nurses, are at risk of suffering aggressive assaults.[5] In fact, the number of WPV incidents reported in health-care facilities accounts for almost one-quarter of the total number of violent incidents reported for all workplaces,[6] making healthcare workers the most vulnerable group to WPV.[7] Naturally, the occurrence of hospital violence may affect the health of physicians and nurses and hinder their professional performance, resulting in a negative impact on patient services and overall health.[8-10]

Instances of such violence can be found in several hospital departments,[11] and many studies have focused on the high incidence of violence in emergency rooms.[12, 13] However, we found a comparable rate of violent incidences in otorhinolaryngology departments. The China Hospital Association 2014 data show that from 2008 to 2012, incidences of severe hospital violence increased from 47.7% to 63.7% of all hospital violence [14] and, upon examining 30 cases of physicians and nurses who were victims

For peer review only - http://bmjopen.bmj.com/site/about/guidelines.xhtml

of fatal WPV in China over the past 10 years, we determined that professionals working in otorhinolaryngology departments account for over 10% of the total.

The main causes of violence towards otorhinolaryngology physicians and nurses include (1) that patients tend to consider otorhinolaryngology-related diseases as less important; (2) that physicians and nurses in this department have limited professional skills compared to those in other departments; and (3) the treatment results of otorhinolaryngological diseases are not ideal, (i.e. many conditions cannot be immediately or completely cured).[15] Thus, considering these dangers, it is clear that the working environment of otorhinolaryngology physicians and nurses should be a concern for hospital administrations, both from a mental health and WPV perspective.

Previous studies have shown that WPV is a contributing factor to employees suffering from mental illnesses such as depression.[16-18] It is well-known that depression is a common illness, and it seems to be more prevalent in high-income countries; the incidence rate of depression among adults in low- to middle-income countries is 11.1%, compared to 14.6% in high-income countries[19, 20]; specifically, the incidences of depression in the United States, Canada, and the UK are 12%, 19.3%, and 15.5%, respectively.[21, 22] In Canada, one in 10 nurses have been found to have depressive symptoms,[23] and in France the situation is even more serious, with one-third of nurse managers suffering from such symptoms.[24] Depressive symptoms can negatively affect job satisfaction, work efficiency, and overall happiness, resulting in a negative attitude toward providing medical treatment. Over the long term, this can not only affect the development of human resources in health fields, but also

For peer review only - http://bmjopen.bmj.com/site/about/guidelines.xhtml

reduce the quality of medical care provided.[9, 10, 25] Research shows that Chinese physicians and nurses experience various psychological issues, with the incidence of depression determined to be 34.2%.[26] In a study based in southern China, (specifically, Guangdong Province), depressive symptoms were found to be associated with WPV, long working hours, shift frequency, and departmental operations.[8] Also notable is the fact that studies have revealed a difference in the proportion of depressive symptoms among nurses in different hospital departments, which strongly suggests that managers should seek to determine the reason for these differences between departments through the use of their employee relations and other suitable processes.[8] Further, it should also be noted that much of the existing research primarily assesses the overall situations of hospitals, with few studies focusing on specialised departments. Of the latter studies, the focus has primarily been placed on the emergency department; however, as mentioned above, the department of otolaryngology witnesses a similar number of violent phenomena as emergency rooms, and more than that found in other departments; therefore, the occurrence of depressive symptoms among otolaryngology staff should be taken seriously.

Otolaryngology is a key department in most hospitals, and has a number of unique characteristics when compared with other departments. First, there are many types of otolaryngology-related diseases, each presenting in unique anatomical locations, and these can affect patients' breathing, speech, smell, and hearing. Many such diseases have long cure times; therefore, providing patients with fast and effective treatment is not always possible. Second, the patient population of an

For peer review only
For peer review only - http://bmjopen.bmj.com/site/about/guidelines.xhtml

otolaryngology department can span a wide range of age groups, resulting in relatively large workloads for physicians and nurses. Third, otolaryngology involves a variety of specialisations, requiring physicians and nurses to engage in continual learning to improve their professional and technological skills.[15, 27]

For these reasons, we hypothesise that the proportion of otolaryngology physicians and nurses suffering from depressive symptoms exceeds that of other hospital departments. While some scholars have studied the influencing factors depressive symptoms have on physicians and nurses in general, few existing studies have assessed WPV-related factors associated with depressive symptoms among otorhinolaryngology physicians and nurses. Consequently, the purpose of this study is: (1) to investigate the prevalence of depressive symptoms among otorhinolaryngology physicians and nurses; and (2) to assess the risk factors that influence depressive symptoms, including WPV-related risk factors and demographic variables, and to present some specific suggestions.

2. METHODS

2.1. Study population

We conducted a retrospective cross-sectional survey of otorhinolaryngologists and nurses from Grade A tertiary hospitals in Heilongjiang Province. Grade A tertiary hospitals are classified in accordance with China's current 'hospital classification management approach', which is designed to implement the division of medical institutions. When a medical institution has over 501 beds, it has the capability to provide high-level, specialised medical and health services in several areas, and also

For peer review only - http://bmjopen.bmj.com/site/about/guidelines.xhtml

to implement higher-level education and scientific research tasks; such hospitals are ranked as 'tertiary'. Further, according to the scoring standard, those that score more than 900 points as general hospitals are categorised into the highest classification level for hospitals in mainland China.[28]

2.2 Data collection

Considering the length of time allotted for this research and the limited time available to clinical staff, we decided to adopt a web-based questionnaire approach, which would help to ensure the integrity and effectiveness of the data collection, and we also chose to apply a convenience sampling method. First, with the help of the chairman of the Heilongjiang Province branch of the Association of Otorhinolaryngology Physicians, we obtained approval from the Heilongjiang Provincial Association of Otorhinolaryngology to conduct the study. The association consequently provided a list of Grade A tertiary hospitals and associated physicians' email addresses, which allowed us to contact otolaryngology directors to seek assistance in acquiring related nurses' email addresses. We then sent these individuals an introductory email with a link to the informed consent form and questionnaire. After the initial e-mail was sent, a reminder e-mail was sent to any physicians and nurses who did not respond initially. Through this process, we obtained 652 valid questionnaires, (total efficiency of 83.6%). The data were collected from January 10, 2017, to February 15, 2017.

2.3. Questionnaire preparation

After obtaining written permission, we sent the respondents a questionnaire that

For peer review only - http://bmjopen.bmj.com/site/about/guidelines.xhtml

was developed by the International Labor Office, (ILO), International Council of Nurses, (ICN), World Health Organization, (WHO), and Public Service International, (PSI) in 2003 with the goal of measuring violence in the workplace.[29] We translated the questionnaire into Mandarin and then back into English to verify the accuracy of the Mandarin version. We then invited the aforementioned experts identified by the otorhinolaryngology association to modify the questions as needed, and then assessed the validity of the contents of the questionnaire, including its suitability for Chinese culture and appropriateness of the translation. These experts included epidemiologists, health-management specialists, public health specialists, health system specialists, otolaryngology experts, and administrative experts, (15 persons). We also conducted a two-week retest reliability check, (Cronbach's $\alpha = 0.87$), by having a group of 42 otorhinolaryngology physicians and nurses from five Grade A tertiary hospitals take the test, and then take it again two weeks later.

The final version of the questionnaire included four parts: (1) the demographic characteristics of the professionals, (gender, age, marital status, years of work experience, education level, occupation, seniority), and workplace-related conditions, number of patients received per day, shift work, number of co-workers for more than half of working hours, and anxiety experienced at work, (which was rated from 1, (absent), to 5, (extremely high),); (2) experience of physical violence in the previous 12 months, (defined as intentional injury to body); (3) experience of nonphysical violence over the previous 12 months, (including verbal abuse, threatening events, and sexual harassment); and (4) depressive symptoms, measured using a Chinese

For peer review only - http://bmjopen.bmj.com/site/about/guidelines.xhtml

version of the Zung Self-Rating Depression Scale, (SDS), which has good reliability. SDS is usually used to measure the level of depressive symptoms in the general population over the previous week, and is widely used in research into WPV and depressive symptoms.[8, 30] Specifically, it facilitates the revealing of subjective feelings using 20 items which are rated in terms of severity using a four-point scale ranging from 1, (absent or minimum), to 4, (high or extremely high). The total score was recorded as the original score, the scores for the 20 items were added together, and finally the resulting score was multiplied by 1.25 to get the standard score. A standard score of 53~62 points represents mild depression, 63~72 points represents moderate depression, and >72 points signifies severe depression. To ensure that the results of this study can be compared with the results of related studies, we chose to use the same classification method as other studies: setting a final score of ⩾ 53 points as indicating the presence of depressive symptoms.[31, 32]

2.4. Data analysis

Descriptive statistical calculations were conducted on the respondents' demographic characteristics and their reported frequency of experiencing depressive symptoms; in addition, a chi-test was used to analyse the correlation between symptoms of depression and the respondents' characteristics, as well as WPV-related factors. Further, logistic regression analysis was used to determine the characteristics and factors, (including years of work experience, education level, seniority, number of patients received per day, number of co-workers for more than half of working hours, anxiety experienced at work, and physical violence), were likely to predict depression.

For peer review only - http://bmjopen.bmj.com/site/about/guidelines.xhtml

SPSS v.19.0 was used to conduct the analysis, (statistical products and services solutions 19.0, 2010, IBM, Armonk, NY, USA). Statistical significance is indicated by $p < 0.05$.

2.5. Ethics statement

All respondents received an informed consent form, which described the goal and method of the data collection, how the data would be handled, and also an assurance of confidentiality. All respondents who gave their informed consent completed the questionnaire. The study protocol was reviewed and approved by the Research Ethics Committee of Harbin Medical University.

3. RESULTS

In Table 1, the demographic characteristics of the study participants are shown. Of these otolaryngology staff, 63.7% were women, almost half, (41.7%), were between 30 and 39 years of age, and three-quarters, (78.1%), were married. The majority of respondents, (69.8%), had at least 10 years of experience in otolaryngology and 78.2% was graduated or specialised. Of the respondents, 19.5% was senior within the department.

Descriptive statistics concerning the depressive symptoms of the participants are presented in Table 2; notably, the table shows that 57.2% of the 652 otorhinolaryngology physicians and nurses had depressive symptoms. Moreover, approximately half. (49.54%). of the respondents worked with one or two colleagues for more than half of their working hours while, of the respondents with depression symptoms, three quarters. (76.41%). had experienced varying degrees of anxiety.

For peer review only - http://bmjopen.bmj.com/site/about/guidelines.xhtml

Additionally, of the respondents who had suffered physical violence, 71.25% had depressive symptoms. We then assessed associations between sample characteristics and depressive symptoms using Pearson chi-square tests and determined that years of work experience, seniority, number of co-workers for more than half of the working hours, anxiety experienced at work, and physical violence are significantly associated with depressive symptoms.

In Table 3, the variables related to depressive symptoms, determined using a multiple logistic model, an adjusted odds ratio, (OR), and a 95% confidence interval, (CI), are shown.Our otorhinolaryngology physicians and nurses who suffered from physical violence, (OR = 1.82, 95% CI = 1.06–3.12), were more likely to suffer from depressive symptoms than those who had not. Moreover, participants with less than one year of experience were also more likely to suffer from depressive symptoms, while those with more experience were progressively less likely to suffer from depressive symptoms. Otorhinolaryngology physicians and nurses who worked alone for more than half of their working hours were more likely to suffer from depressive symptoms than those who worked frequently with other colleagues. Relative to the seniority of otorhinolaryngology physicians and nurses, formal staff and those with junior and intermediate titles showed protective factors for depressive symptoms.

4. DISCUSSION

Our study examined the association between depressive symptoms and WPV-related risk factors in regard to otorhinolaryngology physicians and nurses in northern China. Our results confirm that depressive symptoms are common among

For peer review only - http://bmjopen.bmj.com/site/about/guidelines.xhtml

professionals in this department, with the incidence of depressive symptoms shown to be 57.2%. There is limited research on depression among otorhinolaryngology staff, so we could not directly compare our results with those of other studies; however, these results show a significantly higher prevalence of depressive symptoms than that found among general physicians and nurses in the United States, (11.3%),[33] US nurses (41%),[35] general physicians in the Netherlands, (29%),[34] and Iranian physicians and nurses, (28.76%).[36] Further, this number is also slightly higher than the incidence of depressive symptoms reported among emergency department nurses in Taiwan.[1] In addition, the studies of Yang and Ji et al. reported that the incidence of depressive symptoms among healthcare workers in China across all hospital departments is 45.3% and 47.2%, respectively[37, 38]; however, the incidence of depression differs between departments. Compared with other departments, otorhinolaryngology physicians and nurses have a higher incidence of depressive symptoms than most other departments outside of emergency, and also report higher than normal levels[8, 30]; however, it should be noted that few previous studies have focused on the incidence of depressive symptoms among these professionals in China. Unfortunately, in China, the frequency of violence (which is a major predictor of depression) within otorhinolaryngology department is relatively high due to various factors involving treatment options and professional skill levels[15]; therefore, research into the association between depressive symptoms and WPV-related risk factors is essential for preserving not only the mental health of practitioners, but also for ensuring quality of care.

This study found several factors associated with the occurrence of depressive

For peer review only - http://bmjopen.bmj.com/site/about/guidelines.xhtml

symptoms. First, years of work experience and seniority were identified; the fewer a physician's or nurse's years of work experience, the more likely they are to suffer from depressive symptoms. This has also been confirmed in previous studies on healthcare professionals in China.[39] Less-experienced physicians and nurses often receive insufficient social support, and are more likely be affected by occupational stress.[39, 40] Thus, the mental state of physicians and nurses with few years of experience should be evaluated as early as possible in order to provide them with preventative and proactive psychological support, as well as training opportunities.[41]

Second, considering WPV-related risk factors, number of co-workers for more than half of working hours was found to be a predictor of depression among otorhinolaryngology professionals. The results of the survey showed that, when compared with physicians and nurses who mainly work with two colleagues, those who frequently work alone are more likely to suffer from depressive symptoms; the higher the number of colleagues working together, the lower the risk of depressive symptoms. Working alongside colleagues can help physicians and nurses handle pressure, dissatisfaction, and other emotional fluctuations. Previous studies have also shown that social support can reduce the incidence of depressive symptoms, help individuals manage stress, and promote good mental health.[42] Peer support intervention, in particular, can effectively reduce depressive symptoms, not only because it can be implemented through daily interactions, but also because it can have the same effect as group cognitive behavioural therapy.[43] Support at work is a social-network resource associated with the work environment, and employees at all

For peer review only - http://bmjopen.bmj.com/site/about/guidelines.xhtml

levels can benefit from the support of their colleagues. In the demand-control-support model, it has been highlighted that social support is similar to job control, and may have a buffering or reinforcing effect on individuals' psychological responses; for example, work friendship between colleagues has been reported to be an important social support.[44] Therefore, the working status of medical staff, (e.g. time spent working alone), should be considered in order to strengthen peer support, (as well as team cooperation), and thereby effectively prevent and improve depressive symptoms.

Third, this study revealed that physical violence is an important predictor of incidences of depressive symptoms within the otolaryngology department. Our findings suggest that the risk of physicians and nurses who have suffered physical violence developing depressive symptoms is 1.82 times greater than that for professionals who have not experienced such violence. This is supported by previous surveys, which have also indicated that WPV is a strong predictor of psychological problems such as depressive symptoms.[16] Depression is one of several negative yet common consequences experienced by physicians and nurses who suffer from WPV.[45]Among them, physical violence is more likely to cause physicians and nurses to experience depressive symptoms.[39] The reasons for the high rate of physical violence within otolaryngology departments are varied, but an important factor is that otorhinolaryngology-related diseases are often complex and involve longer courses of treatment, which can lead to patient insomnia, anxiety, and other psychological problems, which can in turn lead to a loss of trust in physicians and nurses. As such, these patients can be prone to adverse emotions.[46] Further, due to the nature of many

For peer review only - http://bmjopen.bmj.com/site/about/guidelines.xhtml

otorhinolaryngology diseases, which affect speech and hearing, patients are often unable to effectively express their dissatisfaction, making them more inclined to choose physical violence to express their discomfort or psychological distress.[47]

Physical violence can also cause psychological damage to physicians and nurses; for example, it can increase levels of anxiety, which can result in poor productivity, lower quality of work, a decline in individual sense of accomplishment, and poor relationships with patients, managers, and colleagues.[12, 47, 48] In addition, physical violence can also have a negative impact on physicians and nurses' social relationships both inside and outside the hospital.[48] Consequently, the relationship between physicians and nurses and their patients should be improved through different measures, including by attaching importance to patients' psychological status during clinical diagnosis and treatment processes, enhancing the communication skills of otorhinolaryngology staff, and improving patients' awareness of their disease and potential treatment options, (including duration and adverse side-effects). Concurrently, medical institutions should adopt a 'zero tolerance' approach to violent incidents and, to reduce the harm caused by physical violence, managers should strengthen the provision of timely psychological comfort and psychological counselling for employees who have experienced physical violence.

In previous studies, the incidence of depressive symptoms was associated with physicians and nurses' workload and shift characteristics[49-53]; however, this study did not find any relevant association between depression and these two factors among otorhinolaryngology professionals, similar to the findings of another study of resident

For peer review only - http://bmjopen.bmj.com/site/about/guidelines.xhtml

physicians in Turkey.[54] Future research should further explore this area.

This study has the following limitations. First, we only investigated otorhinolaryngology physicians and nurses working in Grade A tertiary hospitals in Heilongjiang Province, so the results may not be fully representative of the entire situation in northern China, and cannot be extended to other types of hospitals or departments. Future studies should conduct in-depth exploration of the different types of otorhinolaryngological services at hospitals of different grades. Second, physicians and nurses who were absent from work because of depression were not assessed; thus, the reported rate of depressive symptoms may be lower than the true value. Third, because the respondents were asked to review the events of the past 12 months, the prevalence of violence could have been under-reported as a result of reporting or recall bias or stigma and shame. Fourth, this study focuses on the more distinctive WPV-related factors for this analysis; however, there may be additional factors related to the occurrence of depressive symptoms that could be further explored. Nevertheless, the results of this study indicate that physical violence and mainly working alone are risk factors for depressive symptoms among otorhinolaryngology physicians and nurses, and this provides some basis and direction for future research.

In conclusion, the results show that otorhinolaryngology physicians and nurses in Grade A tertiary hospitals in Heilongjiang Province, China, have a high risk of experiencing depressive symptoms. Of the 652 respondents, 57.2% reported varying degrees of depressive symptoms. Our findings also show that experiencing physical violence, number of co-workers for more than half of working hours, and other

For peer review only - http://bmjopen.bmj.com/site/about/guidelines.xhtml

WPV-related factors are associated with symptoms of depression. Therefore, on one hand, it is clear that medical institutions should pay more attention to otorhinolaryngology. Essential measures include improving communication between medical staff and patients and the adoption of a 'zero tolerance' attitude towards hospital violence. On the other hand, attention should be paid to the construction of team relationships, improving teams' cooperation, and giving full backing to peer support to alleviate depressive symptoms. Finally, more attention should also be paid to lateral violence, (bullying, harassment, physical violence between staff, etc.) to address depressive symptoms caused by such violence. However, as we did not study the impact of lateral violence on depressive symptoms, this represents an avenue of research for future studies.

References

1. Hsieh, H.F., et al., Association among components of resilience and workplace violence‐related depression among emergency department nurses in Taiwan: a cross‐sectional study. Journal of Clinical Nursing, 2016. **25**(17-18): p. 2639-2647.
2. Qiang Ma, An Analysis of the Causes of Frequent Cases of Violent Injuries. National Medical Journal of China, 2014. 94(18): p. 1368-1370.
3. Healthcarefacilities, et al., Violence - Occupational hazards in hospitals. Hospitals, 2002.
4. Simpson, K.R., Incivility, Bullying, and Workplace Violence. Mcn the American Journal of Maternal/child Nursing, 2016. **41**(1): p. 68.
5. Ferri, P., et al., Workplace violence in different settings and among various health professionals in an Italian general hospital: a cross-sectional study. Psychology Research & Behavior Management, 2016. **9**: p. 263-275.
6. ISBN, Framework guidelines for addressing workplace violence in the health

For peer review only - http://bmjopen.bmj.com/site/about/guidelines.xhtml

sector. Geneva Switzerland Ilo, 2002.

7. Sun, P., et al., Workplace Violence against Health Care Workers in North Chinese Hospitals: A Cross-Sectional Survey. International Journal of Environmental Research & Public Health, 2017. **14**(1).

8. Gong, Y., et al., Prevalence of depressive symptoms and work-related risk factors among nurses in public hospitals in southern China: A cross-sectional study. Scientific Reports, 2014. **4**: p. 7109.

9. Landrigan, C.P., et al., Effect of reducing interns' work hours on serious medical errors in intensive care units. New England Journal of Medicine, 2004. **351**(18): p. 1838-1848.

10. Lockley, S.W., et al., Effect of reducing interns' weekly work hours on sleep and attentional failures. New England Journal of Medicine, 2004. **351**(18): p. 1829.

11. Yan, T., et al., Perceived Risk of Violence in Various Hospital Levels and Departments in Urban and Rural China. Iranian Journal of Public Health, 2016. **45**(9): p. 1238-1240.

12. Eslamian, J., A.A. Akbarpoor, and S.A. Hoseini, Quality of work life and its association with workplace violence of the nurses in emergency departments. Iranian Journal of Nursing & Midwifery Research, 2015. **20**(1): p. 56-62.

13. Stene, J., et al., Workplace violence in the emergency department: giving staff the tools and support to report. Permanente Journal, 2015. **19**(2): p. e113-7.

14. Lingling Wang, Chen Wang, Yanlin Cao, Yue Zhao, Yutao Cheng, Lili Zheng., Analysis and thoughts on trend and harm of hospital violence. Chinese Hospitals., 2014(3): p. 4-6.

15. Kai Zhang and Kang Xue, Difficulties and Countermeasures of Young Doctors in the Department of ENT in Primary Hospitals. Medicine and Law, 2016. 8(4): p. 45-47.

16. Aytaç, S. and S. Dursun, The effect on employees of violence climate in the workplace. Work, 2012. **41 Suppl 1**(6): p. 3026.

17. Merecz, D., M. Drabek, and A. Mościcka, Aggression at the

For peer review only - http://bmjopen.bmj.com/site/about/guidelines.xhtml

workplace--psychological consequences of abusive encounter with coworkers and clients. International Journal of Occupational Medicine & Environmental Health, 2009. **22**(3): p. 243.

18. Silva, A.T.C.D., et al., Violence at work and depressive symptoms in primary health care teams: a cross-sectional study in Brazil. Social Psychiatry and Psychiatric Epidemiology, 2015. **50**(9): p. 1-9.

19. Brundtland, G.H., Mental Health: New Understanding, New Hope. Journal of the American Medical Association, 2001. **286**(19): p. 2391-2391.

20. Bromet, E., et al., Cross-national epidemiology of DSM-IV major depressive episode. BMC Medicine, 2011. **9**(1): p. 90-90.

21. Coomber, S., et al., Stress in UK intensive care unit doctors. Bja British Journal of Anaesthesia, 2002. **89**(6): p. 873.

22. Lloyd, S., D. Streiner, and S. Shannon, Burnout, depression, life and job satisfaction among Canadian emergency physicians. Journal of Emergency Medicine, 1994. **12**(4): p. 559-565.

23. Ohler, M.C., M.S. Kerr, and D.A. Forbes, Depression in nurses. The Canadian journal of nursing research = Revue canadienne de recherche en sciences infirmières, 2010. **42**(3): p. 66-82.

24. Nourry, N., et al., Psychosocial and organizational work environment of nurse managers and self-reported depressive symptoms: cross-sectional analysis from a cohort of nurse managers. International Journal of Occupational Medicine and Environmental Health, 2014. **27**(2): p. 252-69.

25. Xiaoying Jiang, Ji Gao, and Xuan Zhang, Correlation analysis about workplace violence and job burnout among the head nurses. Chinese Nursing Management, 2008. 8(3): p. 18-20.

26. Yanqin Yuan, Bo Wang, Xianni Wang, Kuihua Jin., Survey on Mental Health Status of the Medical Staff. Nursing Journal of Chinese People's Liberation Army, 2007. 24(12): p. 22-23.

27. Wei Li, Analysis of Psychological Characteristics of Patients with Otorhinolaryngology and Analysis of Psychological Nursing, Modern

For peer review only - http://bmjopen.bmj.com/site/about/guidelines.xhtml

Diagnosis & Treatment, 2014(2): p. 1428-1429.

28. Yan Li. Interpretation and preparation of the evaluation standard of tertiary general hospital (2011 Edition) by the Ministry of health. Preparation of [C]// "hospital accreditation standards and assessment management interpretation" training paper. 2011.

29. ILO/ICN/WHO/PSI, Workplace Violence in the Health Sector Country Case Study-Questionnaire. 2003: p. 1-14.http://www.who.int/violence_injury_prevention/violence/interpersonal/en/WVquestionnaire.pdf.

30. Gong, Y., et al., Prevalence of Anxiety and Depressive Symptoms and Related Risk Factors among Physicians in China: A Cross-Sectional Study. Plos One, 2014. **9**(7): p. e103242.

31. Zhengyu Wang and Yufen Chi, Self-Rating Depression(SDS). Shanghai Archives of Psychiatry, 1984(2).

32. Zung, W.W.K., A Self-Rating Depression Scale. Archives of General Psychiatry, 1965. **12**(12): p. 63-70.

33. Schwenk, T.L., D.W. Gorenflo, and L.M. Leja, A survey on the impact of being depressed on the professional status and mental health care of physicians. Journal of Clinical Psychiatry, 2008. **69**(4): p. 617-20.

34. Ruitenburg, M.M., H.W. Frings-Dresen Monique, and J.K. Sluiter, The prevalence of common mental disorders among hospital physicians and their association with self-reported work ability: a cross-sectional study. BMC Health Services Research, 2012. **12**(1): p. 1-7.

35. Ruggiero, J.S., Health, work variables, and job satisfaction among nurses. Journal of Nursing Administration, 2005. **35**(5): p. 254-263.

36. Roohafza, H.R., et al., What's the role of perceived social support and coping styles in depression and anxiety? Journal of Research in Medical Sciences the Official Journal of Isfahan University of Medical Sciences, 2014. **19**(10): p. 944-9.

37. Xiaofang Yang and Shaoyun Hu, Related Study between Social Support and

For peer review only - http://bmjopen.bmj.com/site/about/guidelines.xhtml

Coping Style, and Anxiety and Depression of Nurse. China Journal of Health Psychology, 2011. 19(3): p. 300-301.

38. Yongjuan Ji, Lina He, Jing Hao, Guifang Kuang., Correlation study of occupational stress and mental health among medical staff, Occupation and Health, 2015. 31(24): p. 3393-3395.

39. Lijia Tian, Occupational stress and its relationship to depressive symptoms and anxiety disotder among ophthalmologists in Shanghai.2013, Fudan University.

40. Raffaelli, M., et al., Stress, Social Support, and Depression: A Test of the Stress-Buffering Hypothesis in a Mexican Sample. Journal of Research on Adolescence, 2013. **23**(2): p. 283–289.

41. Hassan, T.M., et al., A postal survey of doctors' attitudes to becoming mentally ill. 2009. **9**(4): p. 327-332.

42. Hsieh, H.F., et al., Association among components of resilience and workplace violence-related depression among emergency department nurses in Taiwan: a cross-sectional study. Journal of Clinical Nursing, 2016. **25**(17-18): p. 2639-2647.

43. Piette, J.D., Efficacy of peer support interventions for depression: a meta-analysis. General Hospital Psychiatry, 2011. **33**(1): p. 29.

44. Qian Shi, Xiaobing Shu, and Yuyue Luo, The job demand-control-support model a review of empirical research. Advances in Psychological Science, 2010. **18**(4): p. 655-663.

45. Alexy, E.M. and J.A. Hutchins, Workplace violence: a primer for critical care nurses. Critical Care Nursing Clinics of North America, 2006. **18**(3): p. 305-12.

46. Yu Liu and Weiping He, Ethical Ｒeflections on Medical Dispute in the Department of Otorhinolaryngology. Chinese Medical Ethics, 2016. 29(5): p. 777-779.

47. Roldán, G.M., et al., Violence at work and its relationship with burnout, depression and anxiety in healthcare professionals of the emergency services.

For peer review only

Health, 2013. **05**(2): p. 193-199.

48. Y, D., et al., Bullying among nurses and its effects. International Nursing Review, 2009. **56**(4): p. 504-511.

49. Lee, H.E., H.R. Kim, and J.S. Park, Work-related risk factors for workplace violence among Korean employees. Journal of Occupational Health, 2014. **56**(1): p. 12.

50. Virtanen, M. and M. Kivimäki, Saved by the bell: does working too much increase the likelihood of depression? Expert Review of Neurotherapeutics, 2012. **12**(5): p. 497-499.

51. Bannai, A. and A. Tamakoshi, The association between long working hours and health: a systematic review of epidemiological evidence. Scandinavian Journal of Work Environment & Health, 2014. **40**(1): p. 5-18.

52. Oliveira, F.P.D., et al., Symptoms of depression and intervening factors among nurses of emergency hospital services. Acta Paulista De Enfermagem, 2015. **28**(3): p. 209-215.

53. Ferri, P., et al., The impact of shift work on the psychological and physical health of nurses in a general hospital: a comparison between rotating night shifts and day shifts. Risk Management & Healthcare Policy, 2016. **9**: p. 203-211.

54. Demir, F., et al., [The prevalence of depression and its associated factors among resident doctors working in a training hospital in Istanbul]. Türk psikiyatri dergisi = Turkish journal of psychiatry, 2007. **18**(1): p. 31.

Author contributions

H.F., X.Z., H.Y. drafted the manuscript; J.M. designed the study; P.S., Y.L., K.J., and P.L. collected the data; J.M., M.L., H.Q., and Q.W. analyzed the data; J.M. contributed to the manuscript's revision. All authors approved the final manuscript for publication.

For peer review only - http://bmjopen.bmj.com/site/about/guidelines.xhtml

Table 1. Participant demographic characteristics (N = 652).

Characteristic	n	%
**Gender**		
Male	237	36.3%
Female	415	63.7%
**Age**		
≤29	214	32.8%
30–39	272	41.7%
≥40	166	25.5%
**Marital status**		
Unmarried	130	19.9%
Married	509	78.1%
Divorced	9	1.4%
Widowed	1	0.2%
Other	3	0.5%
**Years of work experience**		
<1	76	11.7%
1-10	379	58.1%
11-20	126	19.3%
21-30	61	9.4%

For peer review only - http://bmjopen.bmj.com/site/about/guidelines.xhtml

>30	10	1.5%
**Seniority**		
Senior	127	19.5%
Intermediate	237	36.3%
Primary	168	25.8%
No title (formal doctor)	65	10.0%
Interns (resident training in hospital or post-graduate)	55	8.4%
**Education level**		
Graduate and above	130	19.9%
Undergraduate	380	58.3%
Community college	97	14.9%
Vocational school	40	6.1%
Other	5	0.8%
**Occupation**		
Physician	379	58.1%
Nurse	273	41.9%

For peer review only

For peer review only - http://bmjopen.bmj.com/site/about/guidelines.xhtml

Table 2. Descriptive statistics associated with depressive symptoms of the participants.

Variables	Non-depressive tendency N = 279(42.8%) n (%)	Depressive tendency N = 373 (57.2%) n (%)	x^2	P
**Gender**			0.01	0.92
Male	102 (36.56%)	135 (36.19%)		
Female	177 (63.44%)	238 (63.81%)		
**Age**				
≤29	94 (33.69%)	120 (32.17%)	0.20	0.91
30–39	114 (40.86%)	158 (42.36%)		
≥40	71 (25.45%)	95 (25.47%)		
**Marital status**			4.92	0.30
Unmarried	49 (17.56%)	81 (21.72%)		
Married	226 (81.00%)	283 (75.88%)		
Divorced	2 (0.72%)	7 (1.88%)		
Widowed	0 (0%)	1 (0.27%)		
Other	2 (0.72%)	1 (0.27%)		
**Years of work experience**			10.26	0.04
<1	33 (11.83%)	43 (11.53%)		
1-10	163 (58.42%)	216 (57.91%)		

For peer review only

For peer review only - http://bmjopen.bmj.com/site/about/guidelines.xhtml

11-20	48 (17.20%)	78 (20.91%)		
21-30	26 (9.32%)	35 (9.38%)		
>30	9 (3.23%)	1 (0.27%)		
**Seniority**			14.00	0.01
Senior	52 (18.64%)	75 (20.11%)		
Intermediate	89 (31.90%)	148 (39.68%)		
Primary	69 (24.73%)	99 (26.54%)		
No title (formal doctor)	39 (13.98%)	26 (6.97%)		
Interns (resident training in hospital or post-graduate)	30 (10.75%)	25 (6.70%)		
**Education level**			7.32	0.12
Graduate and above	52 (18.64%)	78 (20.91%)		
Undergraduate	174 (62.37%)	206 (55.23%)		
Community college	31 (11.11%)	66 (17.69%)		
Vocational school	20 (7.17%)	20 (5.36%)		
Others	2 (0.72%)	3 (0.80%)		
**Occupation**			0.45	0.50
Physicians	158(56.63%)	221(59.25%)		
Nurses	121(43.37%)	152(40.75%)		
**Number of patients received per day**			1.02	0.91

For peer review only - http://bmjopen.bmj.com/site/about/guidelines.xhtml

1-20	122 (43.73%)	154 (41.29%)		
21-30	83 (29.75%)	116 (31.10%)		
31-40	39 (13.98%)	55 (14.75%)		
41-50	13 (4.66%)	22 (5.90%)		
>51	22 (7.89%)	26 (6.97%)		
**Shift work**			0.01	0.92
Yes	222 (79.57%)	298 (79.89%)		
No	57 (20.43%)	57 (15.28%)		
**Number of coworkers for more than half of the working hours**			13.62	0.01
1	17 (6.09%)	40 (10.72%)		
2	107 (38.35%)	159 (42.63%)		
3-5	97 (34.77%)	129 (34.58%)		
6-10	35 (12.54%)	32 (8.58%)		
>10	23 (8.24%)	13 (3.49%)		
**Anxiety experienced at work**			9.54	0.04
Absent	89 (31.90%)	88 (23.59%)		
Low	52 (18.64%)	61 (16.35%)		
Moderate	74 (26.52%)	105 (28.15%)		
High	23 (8.24%)	38 (10.19%)		

For peer review only - http://bmjopen.bmj.com/site/about/guidelines.xhtml

Extremely high	41 (14.70%)	81 (21.72%)		
**Physical Violence**			3.34	0.01
Yes	23 (8.24%)	57 (15.28%)		
No	256 (91.76%)	316 (84.72%)		
**Non-Physical Violence**			3.43	0.06
Yes	91 (32.62%)	148 (39.68%)		
No	188 (67.38%)	225 (60.32%)		

For peer review only

For peer review only - http://bmjopen.bmj.com/site/about/guidelines.xhtml

Table 3. Multiple logistic regression of depressive and non-depressive symptoms.

	OR	95% CI	P
**Years of work experience**			
<1	1.00	Reference	
1-10	0.04	0.01–0.39	0.01
11-20	0.06	0.01–0.52	0.01
21-30	0.06	0.01–0.52	0.01
>30	0.07	0.01–0.66	0.02
**Education level**			
Graduate and above	1.00	Reference	
Undergraduate	1.32	0.20–8.89	0.78
Community college	1.38	0.21–8.85	0.74
Vocational school	0.67	0.10–4.46	0.68
Others	1.45	0.21–10.13	0.71
**Seniority**			
Senior	1.00	Reference	
Intermediate	0.37	0.16–0.85	0.02
Primary	0.35	0.17–0.73	0.01
No title (formal doctor)	0.48	0.23–0.99	0.04
Interns (resident	0.92	0.40–2.15	0.85

For peer review only
For peer review only - http://bmjopen.bmj.com/site/about/guidelines.xhtml

training in hospital or post-graduate)			
**Number of patients received per day**			
1-20	1.00	Reference	
21-30	1.03	0.52–2.06	0.93
31-40	0.98	0.48–1.98	0.94
41-50	0.88	0.40–1.93	0.75
>51	0.71	0.27–1.87	0.49
**Number of coworkers for more than half of the working hours**			
1	1.00	Reference	
2	0.27	0.11–0.70	0.01
3-5	0.41	0.20–0.89	0.02
6-10	0.41	0.19–0.91	0.03
>10	0.73	0.30–1.74	0.47
**Anxiety experienced at work**			
Absent	1.00	Reference	
Low	1.84	1.09–3.09	0.02

For peer review only - http://bmjopen.bmj.com/site/about/guidelines.xhtml

Moderate	1.61	0.91–2.83	0.10
High	1.30	0.78–2.15	0.31
Extremely high	1.13	0.58–2.21	0.72
**Physical Violence**			
Yes	1.82	1.06–3.12	0.03
No	1.00	Reference	

For peer review only - http://bmjopen.bmj.com/site/about/guidelines.xhtml

STROBE 2007 (v4) Statement—Checklist of items that should be included in reports of *cross-sectional studies*

Section/Topic	Item #	Recommendation	Reported on page #
**Title and abstract**	1	(*a*) Indicate the study's design with a commonly used term in the title or the abstract	1
		(*b*) Provide in the abstract an informative and balanced summary of what was done and what was found	3-4
**Introduction**			
Background/rationale	2	Explain the scientific background and rationale for the investigation being reported	6-8
Objectives	3	State specific objectives, including any prespecified hypotheses	8-9
**Methods**			
Study design	4	Present key elements of study design early in the paper	9
Setting	5	Describe the setting, locations, and relevant dates, including periods of recruitment, exposure, follow-up, and data collection	9-13
Participants	6	(*a*) Give the eligibility criteria, and the sources and methods of selection of participants	9-10
Variables	7	Clearly define all outcomes, exposures, predictors, potential confounders, and effect modifiers. Give diagnostic criteria, if applicable	10-12
Data sources/ measurement	8*	For each variable of interest, give sources of data and details of methods of assessment (measurement). Describe comparability of assessment methods if there is more than one group	10-12
Bias	9	Describe any efforts to address potential sources of bias	*10*

For peer review only

Study size	10	Explain how the study size was arrived at	10
Quantitative variables	11	Explain how quantitative variables were handled in the analyses. If applicable, describe which groupings were chosen and why	10-12
Statistical methods	12	(*a*) Describe all statistical methods, including those used to control for confounding	12-13
		(*b*) Describe any methods used to examine subgroups and interactions	12-13
		(*c*) Explain how missing data were addressed	10
		(*d*) If applicable, describe analytical methods taking account of sampling strategy	13
		(*e*) Describe any sensitivity analyses	
**Results**			
Participants	13*	(a) Report numbers of individuals at each stage of study—eg numbers potentially eligible, examined for eligibility, confirmed eligible, included in the study, completing follow-up, and analysed	10
		(b) Give reasons for non-participation at each stage	
		(c) Consider use of a flow diagram	
Descriptive data	14*	(a) Give characteristics of study participants (eg demographic, clinical, social) and information on exposures and potential confounders	13/26-27
		(b) Indicate number of participants with missing data for each variable of interest	13
Outcome data	15*	Report numbers of outcome events or summary measures	14
Main results	16	(*a*) Give unadjusted estimates and, if applicable, confounder-adjusted estimates and their precision (eg, 95% confidence interval). Make clear which confounders were adjusted for and why they were included	14/28-34
		(*b*) Report category boundaries when continuous variables were categorized	28-34

For peer review only

		(*c*) If relevant, consider translating estimates of relative risk into absolute risk for a meaningful time period	
Other analyses	17	Report other analyses done—eg analyses of subgroups and interactions, and sensitivity analyses	
**Discussion**			
Key results	18	Summarise key results with reference to study objectives	14-15
Limitations	19	Discuss limitations of the study, taking into account sources of potential bias or imprecision. Discuss both direction and magnitude of any potential bias	19
Interpretation	20	Give a cautious overall interpretation of results considering objectives, limitations, multiplicity of analyses, results from similar studies, and other relevant evidence	15-19
Generalisability	21	Discuss the generalisability (external validity) of the study results	19-20
**Other information**			
Funding	22	Give the source of funding and the role of the funders for the present study and, if applicable, for the original study on which the present article is based	4-5

*Give information separately for cases and controls in case-control studies and, if applicable, for exposed and unexposed groups in cohort and cross-sectional studies.

Note: An Explanation and Elaboration article discusses each checklist item and gives methodological background and published examples of transparent reporting. The STROBE checklist is best used in conjunction with this article (freely available on the Web sites of PLoS Medicine at http://www.plosmedicine.org/, Annals of Internal Medicine at http://www.annals.org/, and Epidemiology at http://www.epidem.com/). Information on the STROBE Initiative is available at www.strobe-statement.org.

For peer review only
